# Evidence of a putative CO_2_ delivery system to the chromatophore in the photosynthetic amoeba *Paulinella*

**DOI:** 10.1111/1758-2229.13304

**Published:** 2024-06-23

**Authors:** Arwa Gabr, Timothy G. Stephens, John R. Reinfelder, Pinky Liau, Victoria Calatrava, Arthur R. Grossman, Debashish Bhattacharya

**Affiliations:** ^1^ Graduate Program in Molecular Bioscience and Program in Microbiology and Molecular Genetics Rutgers University New Brunswick New Jersey USA; ^2^ Department of Biochemistry and Microbiology Rutgers University New Brunswick New Jersey USA; ^3^ Department of Environmental Sciences Rutgers University New Brunswick New Jersey USA; ^4^ Department of Plant Biology The Carnegie Institution for Science Stanford California USA

## Abstract

The photosynthetic amoeba, *Paulinella* provides a recent (ca. 120 Mya) example of primary plastid endosymbiosis. Given the extensive data demonstrating host lineage‐driven endosymbiont integration, we analysed nuclear genome and transcriptome data to investigate mechanisms that may have evolved in *Paulinella micropora* KR01 (hereinafter, KR01) to maintain photosynthetic function in the novel organelle, the chromatophore. The chromatophore is of α‐cyanobacterial provenance and has undergone massive gene loss due to Muller's ratchet, but still retains genes that encode the ancestral α‐carboxysome and the shell carbonic anhydrase, two critical components of the biophysical CO_2_ concentrating mechanism (CCM) in cyanobacteria. We identified KR01 nuclear genes potentially involved in the CCM that arose via duplication and divergence and are upregulated in response to high light and downregulated under elevated CO_2_. We speculate that these genes may comprise a novel CO_2_ delivery system (i.e., a biochemical CCM) to promote the turnover of the RuBisCO carboxylation reaction and counteract photorespiration. We posit that KR01 has an inefficient photorespiratory system that cannot fully recycle the C_2_ product of RuBisCO oxygenation back to the Calvin‐Benson cycle. Nonetheless, both these systems appear to be sufficient to allow *Paulinella* to persist in environments dominated by faster‐growing phototrophs.

## Graphical abstract

The photosynthetic amoeba, *Paulinella* provides a model for understanding plastid primary endosymbiosis. We analysed genomic data to understand how *Paulinella* delivers CO_2_ to the site of fixation in the photosynthetic organelle, the chromatophore. We discovered a putative CO_2_ delivery system (i.e., a biochemical carbon concentrating mechanism, CCM) that may promote turnover of the RuBisCO carboxylation reaction and counteract photorespiration.

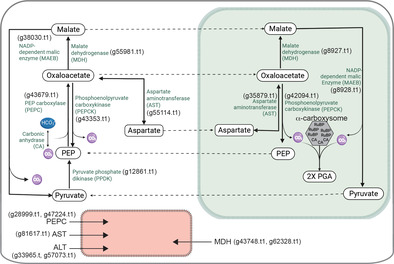

## INTRODUCTION

Photosynthetic *Paulinella*, a rhizarian amoeba and a recent example of primary plastid endosymbiosis (ca. 120 Mya), represents a model system for studying the earlier stages of organellogenesis relative to the much longer evolutionary history (>1.6 Bya) of the Archaeplastida (Bhattacharya et al., 2023; Delaye et al., 2016; Gabr et al., 2020; Lhee et al., 2019; Nowack et al., 2008; Stephens et al., 2021; Van Etten et al., 2023). The photosynthetic organelle in *Paulinella*, referred to as the chromatophore, supplies its host with energy in the form of reduced carbon, allowing this previously heterotrophic lineage to transition to a fully photoautotrophic lifestyle (Kies, 1974; Kies & Kremer, 1979). However, *Paulinella* is extremely slow growing, with a doubling time of 5–7 days in replete culture medium (Zhang et al., 2017). In addition, these cells can only grow under low light (10–20 μmol photons m^−2^ s^−1^) (Zhang et al., 2017), whereas free‐living *Synechococcus* spp. (putative chromatophore ancestor, and member of the α‐cyanobacterial clade) have a maximum doubling rate of >1.4 times/day and tolerate a much broader range of light intensities (30–2000 μmol photons m^−2^ s^−1^) (Kana & Glibert, 1987). A previous study showed that all genes necessary for photosynthesis are present in either (or both) the chromatophore and nuclear genomes of *Paulinella*, suggesting that the loss of photosynthesis‐related genes is unlikely to explain the slow growth phenotype (Gabr, Zournas, et al., 2022).

Factors potentially limiting the growth rate of *Paulinella* include poor metabolic connectivity (metabolite exchange) between the chromatophore and host that is exacerbated by exposure to high‐intensity light. This may impair overall metabolism and cause elevated intracellular redox potentials and the formation of reactive oxygen species that result in damage to *Paulinella* cellular processes (Stephens et al., 2021; Zhang et al., 2017). In addition, the transport of host, cytoplasmically synthesized proteins into the chromatophore may be suboptimal. ‘Long’ cytoplasmically‐synthesized proteins (i.e., >268 amino acids [aa] in length) in *Paulinella* that are targeted to the chromatophore contain a unique ~200 aa *N*‐terminal extension (known as a chromatophore transit peptide [crTP]) that can target proteins to the chloroplast in tobacco (Singer et al., 2017). Proteins harbouring this N‐terminal extension are therefore most likely to be chromatophore‐targeted. In contrast, ‘short’ chromatophore‐targeted proteins (<90 aa in length; lacking a crTP) are transported across the chromatophore membrane via a separate mechanism that appears to involve the secretory pathway (Nowack & Grossman, 2012). Finally, analysis of enriched insoluble protein fractions derived from the chromatophore inner membrane does not provide evidence of canonical, nucleus‐encoded, organelle‐targeted metabolite transporters, as is the case for Archaeplastida (Karkar et al., 2015). Rather, short chromatophore‐targeted orphan proteins (related to octotricopeptide repeats) may modulate membrane permeability (Oberleitner et al., 2020), in which case, metabolite exchange between the host and endosymbiont may have low efficiency.

Given these observations, it is of interest to investigate mechanisms that may have arisen (and are likely still evolving) in *Paulinella* to support its obligate photosynthetic lifestyle and provide insights into its growth phenotype. These innovations will most likely occur in the host nuclear, rather than the chromatophore genome, which is undergoing Muller's ratchet, resulting in massive genome reduction (Stephens et al., 2021). Past work also shows that key chromatophore genes involved in photosynthesis do not show light regulation (Zhang et al., 2017). Therefore, there is a presumptive added burden on the host to balance photosynthesis and photorespiration in an organelle which encodes genes that may have unregulated expression (see below).

In this regard, a key constraint faced by photoautotrophs is the need to efficiently fix CO_2_ via the Calvin‐Benson cycle of the C_3_ pathway (von Caemmerer & Evans, 2010; Yamori et al., 2014). The enzyme ribulose‐1,5‐bisphosphate carboxylase/oxygenase (RuBisCO) is central to this cycle and incorporates CO_2_ into 3‐phosphoglyceric acid (3‐PGA) (Johnson, 2016), which is reduced to dihydroxyacetone phosphate/phosphoglyceraldehyde. These products can be converted into glucose, other sugars, and fixed carbon polymers that drive many downstream metabolic processes (e.g., amino acid and lipid biosynthesis), or are used for energy storage (starch, triacylglyceride). However, cyanobacterial RuBisCOs have very low affinities for CO_2_ (K_CO2_ >200 μM; Moroney & Somanchi, 1999), and O_2_ in the atmosphere competes with CO_2_ for binding to the active site of this enzyme (Giordano et al., 2005). RuBisCO can catalyse the oxygenation of ribulose‐1,5‐bisphosphate (oxygenase activity) converting it into 3‐PGA and 2‐phosphoglycolate, with the latter channelled into the photorespiratory C_2_ cycle. Photorespiration leads to a loss of fixed carbon and energy, resulting in a ~30% decrease in photosynthetic efficiency (Sage, 2004). This issue is most severe under high light (HL), elevated temperature, and desiccation conditions. In addition, the 2‐phosphoglycolate synthesized due to RuBisCO oxygenase activity is toxic, inhibiting enzymes associated with photosynthetic metabolism, and is removed from the cell by an energy‐intensive multi‐step enzymatic reaction (Sheen, 1999). To counteract photorespiration, various mechanisms (known as the CO_2_ concentrating mechanism [CCM]) have evolved independently in different lineages, including algae, to concentrate CO_2_ in the subcellular compartment that houses RuBisCO (Brautigam & Gowik, 2016; Kupriyanova et al., 2023; Schluter & Weber, 2020; Wei et al., 2019). These are referred to as biophysical or biochemical CCMs based on how CO_2_ is delivered to the site of RuBisCO. The former functions by transporting inorganic carbon from the exterior to the interior of the cell, concentrating bicarbonate (HCO_3_^−^) in the compartment that houses RuBisCO and converting it to CO_2_ (substrate of RuBisCO) via the highly efficient enzyme, carbonic anhydrase (CA; Tsuji et al., 2017). This mechanism increases the rate of photosynthetic CO_2_ fixation and is essential to the biophysical CCM of cyanobacteria and microalgae (Badger & Price, 2003; Kupriyanova et al., 2023; Price et al., 1992; Wei et al., 2019). In contrast, biochemical CCMs, present in C_4_ plants, temporarily fix CO_2_ into a 4‐carbon organic intermediate (e.g., malate). These intermediates are transported to the site of RuBisCO where they are decarboxylated to produce pyruvate, concentrated CO_2_, and the reductant [NAD(P)H] (Hennacy & Jonikas, 2020), which allows for efficient CO_2_ fixation.

*Paulinella* inherited a biophysical CCM from its α‐cyanobacterial ancestor that relied on an α‐carboxysome (i.e., an icosahedral protein shell surrounding RuBisCO and a CA; Hennacy & Jonikas, 2020) to enhance the carboxylase activity of RuBisCO (Cai et al., 2015; Rae et al., 2013). Interestingly, the complete operon (referred to as *cso*) encoding components of the ancestral α‐carboxysome (including form 1A RubisCO) of proteobacterial origin that was acquired via horizontal gene transfer (HGT) in the α‐cyanobacterial ancestor is present in the *Paulinella* chromatophore genome (Marin et al., 2007). Proteomic analysis of the chromatophore demonstrates that α‐carboxysome proteins are highly abundant in the organelle fraction (Singer et al., 2017). Key to the activity of this CCM is the production of CO_2_ by a CA (*csoSCA*) which is encoded as part of the *cso* operon on the chromatophore genome. The only known nucleus‐encoded component of the α‐carboxysome that arose via endosymbiotic gene transfer (EGT) from the chromatophore genome (Nowack et al., 2011) is CsoS4A (peptide A). This is a pentameric carboxysome shell protein that supports the carboxysome structure by capping its 12 vertices and may function as a CO_2_ leakage barrier (Cai et al., 2009). This protein in KR01 is 110 amino acids long, and although not identified in the proteomic analysis of the chromatophore (Singer et al., 2017), may be small enough in size to enter the organelle via the secretory pathway (Nowack & Grossman, 2012). Differential regulation of this nuclear gene provides a potential marker of carboxysome functionality in KR01 because deletion of *csoS4A* in the sulfur‐oxidizing chemoautotroph *Halothiobacillus neapolitanus* reduces the catalytic activity of RubisCO in isolated carboxysomes, even though the RuBisCO does not appear to be compromised, potentially because of leakage of CO_2_ from the carboxysome (Cai et al., 2009).

Given these findings, we searched the nuclear gene inventory of our model species *Paulinella micropora* strain KR01 (hereinafter, KR01) for clues as to whether this organism has a ‘rudimentary’ CCM and if so, how the CCM may function in this species. Does KR01 rely solely on an ancestral biophysical CCM encoded by the *cso* operon, or has a biochemical CCM (analogous to C_4_ metabolism) evolved to improve CO_2_ delivery to the chromatophore and carboxysome? We focused here on the latter possibility, and analysis of KEGG pathways in the predicted proteome of KR01‐identified genes that may be involved in a biochemical CCM. We do not expect, however, that the putative KR01 biochemical CCM would strictly follow the canonical version present in algae and plants (e.g., Kroth et al., 2008; Ouyang et al., 2013; Reinfelder et al., 2000; Stutz et al., 2014; Xu et al., 2012) due to its earlier stage of evolution. To explore the predicted functions of target genes identified in our study, we generated RNA‐seq data from cultures incubated under different light and CO_2_ conditions for 6 h. Our work relied on polyA‐selected cDNA libraries, therefore we have no data regarding the differential expression of chromatophore‐encoded genes, such as in the *cso* operon. Our results suggest that a novel, host‐derived CO_2_ delivery system may have evolved in KR01, but some key details are still unknown or uncertain.

## EXPERIMENTAL PROCEDURES

### Cell culture and treatments

The *P. micropora* KR01 culture used in this study was obtained from Prof. Hwan Su Yoon (Sungkyunkwan University) whose group isolated the cells in 2009 from Mangae Jeosuji (Reservoir), Chungnam Province, South Korea. The KR01 cells were grown at 26°C in Fernbach flasks containing 0.3 L DY‐V medium (Andersen et al., 2005), using a 16 h:8 h light/dark cycle (light on/off) at a light intensity of ~15–20 μmol photons m^−2^ s^−1^ (control conditions; *n* = 4). Differential gene transcription was examined after incubating cells under various light, CO_2_, and pH conditions for 6 h. For high light treatment (HL; *n* = 4), cells were immediately shifted to an intensity of 150 μmol photons m^−2^ s^−1^ at the beginning of the light cycle. This light intensity was chosen because it is high enough to elicit a high‐light‐specific response, but not sufficient to cause cell death in the short‐term. Note that the medium for control and HL treatments was buffered at pH 6.8 with 10 mM MES (2‐(N‐morpholino)ethanesulfonic acid) and pre‐equilibrated with air (pCO_2_ ≈10^−3.4^ atm) prior to the addition of cells. For these treatments, the concentration of dissolved CO_2_ (CO_2aq_) was 14 μM and of HCO_3_^−^ was 38 μM (see calculations below). For the elevated CO_2_ treatment (BC; *n* = 4), the medium was supplemented with sodium bicarbonate (NaHCO_3_; final concentration of 5 mM), but was not pre‐equilibrated with air, and cells were incubated under control (low) light conditions. Bicarbonate supplementation resulted in a nearly 8‐fold increase in the concentration of dissolved CO_2_ ([CO_2aq_] = 110 μM, see below). For the high light plus elevated CO_2_ (NaHCO_3_) treatment (HLBC; *n* = 4), cells were incubated under both high light and supplemental bicarbonate conditions as described above. Because the addition of bicarbonate caused the pH of the culture medium to increase from 6.8 to 8.0, we also conducted treatments (pH; *n* = 4) in which culture media was adjusted to pH 8.0 using 1 M NaOH and then pre‐equilibrated with air prior to the addition of cells. In these treatments, the concentration of CO_2_ was the same as in the control and HL treatments (14 μM) and the concentration of HCO_3_^−^ was 600 μM. In all experiments, cells were incubated under the various treatment conditions for 6 h before RNA was harvested. Given the slow rate of KR01 photosynthesis, the carbonate system would not have changed significantly during these short‐term incubations. For example, with a growth rate of 0.12 d^−1^ and a starting cell biomass of 200 μmol carbon/L, KR01 would remove less than 10 μM of dissolved inorganic carbon during a 6‐h incubation.

### Calculation of CO_2aq_ and HCO_3_ ^−^ concentrations

Control and HL treatment (pH = 6.8, open system):

(1) $$\left[CO_{2\mathrm{aq}}\right]=\mathrm{pC}O_{2}\times \mathrm{KH}$$

$$\begin{array}{rcl} \left[{\mathrm{CO}}_{\text{2aq}}\right] & = & \left(10^{-3.4}\;\mathrm{atm}\right)\left(10^{-1.47}\;M\;{\mathrm{atm}}^{-1}\right)\\ & = & 1.35\times 10^{-5}\;M\approx 14\;\mathrm{\mu M} \end{array}$$

(2) $$\left[\mathrm{HC}O_{3}\right]=\left(\mathrm{pC}O_{2}\times \mathrm{KH}\times \mathrm{Ka}1\right)/\left(10^{\text{−}\text{pH}}\right)$$

$$\begin{array}{rcl} \left[\mathrm{HC}O_{3}\right] & = & \frac{\left(10^{-3.4}\;\mathrm{atm}\right)\times \left(\frac{10^{-1.47}M}{\mathrm{atm}}\right)\times \left(10^{-6.35}\right)}{10^{-6.8}}\\ & = & 3.80\times 10^{-5}M=\sim 38\;\mathrm{\mu M} \end{array}$$

BC treatment (pH = 8.0, closed system):

(3) $$\left[{\mathrm{CO}}_{\text{2aq}}\right]=\mathrm{Ci}/\left(1+K_{\text{a1}}{\left[H^{+}\right]}^{-1}+K_{\text{a1}}K_{\text{a2}}{\left[H^{+}\right]}^{-2}\right)$$

$$\begin{array}{rcl} \left[{\mathrm{CO}}_{\text{2aq}}\right] & = & 5\times 10^{-3}M/(1+10^{-6.35}/10^{-8}\\ & & +\;10^{-16.68}/10^{-16})\\ & = & 1.09\times 10^{-4}\;M\approx 110\;\mathrm{\mu M} \end{array}$$

(4) $$\left[{{\mathrm{HCO}}_{3}}^{-}\right]=\mathrm{Ci}/\left(\left[H^{+}\right]{K_{\text{a1}}}^{-1}+1+K_{\text{a2}}{\left[H^{+}\right]}^{-1}\right)$$

$$\begin{array}{rcl} \left[{{\mathrm{HCO}}_{3}}^{-}\right] & = & 5\times 10^{-3}\;M/(10^{-8}/10^{-6.35}+1\\ & & +\;10^{-10.33}/10^{-8})\\ & = & 4.87\times 10^{-3}\;M\approx 4.9\;\mathrm{mM} \end{array}$$

In Equations (3) and (4), Ci is the concentration of total dissolved inorganic carbon.

Elevated pH treatment (pH = 8.0, open system):

$$\begin{array}{rcl} \left[{\mathrm{CO}}_{\text{2aq}}\right] & = & \left(10^{-3.4}\;\mathrm{atm}\right)\left(10^{-1.47}\;M\;{\mathrm{atm}}^{-1}\right)\\ & = & 1.35\times 10^{-5}\;M\approx 14\;\mathrm{\mu M} \end{array}$$

$$\begin{array}{rcl} \left[\mathrm{HC}O_{3}\right] & = & \frac{\left(10^{-3.4\;\mathrm{atm}}\right)\times \left(\frac{10^{-1.47}M}{\mathrm{atm}}\right)\times \left(10^{-6.35}\right)}{10^{-8.0}}\\ & = & 6.03\times 10^{-4}M=\sim 600\;\mathrm{\mu M} \end{array}$$

### RNA extraction and RNA‐seq

Cells were centrifuged at 4000 rpm for 10 min at 4°C. The resulting pellets were frozen in liquid nitrogen and stored at −80°C until RNA was extracted using a Qiagen RNeasy Plant Mini Kit following the manufacturer's instructions (Qiagen, Hilden, Germany). Total RNA was eluted with 50 μL RNAase‐free water and quantified using a Qubit. Library construction and RNA‐sequencing on a HiSeq instrument 2500 (2 × 150 bp reagents) was done by Genewiz.

### Differential gene expression analysis

RNA‐seq reads were trimmed using the CLC Genomics Workbench 8.5.1 (default settings; Qiagen, Hilden, Germany) and individually mapped to the reference genome using this program package. Only the raw “unique exon read” counts were used for downstream analyses (raw count data can be found in Supplemental Table 1). Differential expression analysis was done in RStudio 2021.9.2.382 (Team, 2022) using the DESeq2 package version 1.34.0 (Love et al., 2014). The DESeq2 normalized counts were used for sample‐level QC using principal component analysis (PCA) and hierarchical clustering (Supplemental Figure 1). Based on the sample‐level QC results, one sample from each treatment (control1, pH 1, HL3, and BC4) was removed because they represented outliers that differed significantly from the other replicate samples; these samples were excluded from downstream analysis. Significant DEGs between treatments were identified using DESeq2. Only genes with a Benjamini‐Hochberg adjusted *p*‐value <0.05 were considered for downstream analysis (Love et al., 2014).

### Identification of biochemical CCM‐related genes

KAAS (KEGG Automatic Annotation Server: http://www.genome.jp/kegg/kaas/) (Moriya et al., 2007) was used to assign KEGG Orthology (KO) numbers (K numbers) to the proteins predicted in the KR01 nuclear genome. The assigned KO numbers were used with the KEGG pathway mapper (Kanehisa & Sato, 2020) to generate metabolic maps from the predicted proteins in KR01. Initially, genes related to a biochemical CCM in KR01, identified using KEGG map00710 (*Carbon fixation in photosynthetic organisms*), were extracted and manually validated.

### Functional annotation of KR01 proteins

Functional annotation of all expressed genes was done using BLASTp against the nr database (Lhee et al., 2021). GO terms were assigned to the expressed genes using PANNZER2 (Toronen et al., 2018). DEGs with an adjusted *p*‐value <0.05 were tested for enrichment of GO terms using the topGO R package (Alexa & Rahnenführer, 2010), with all predicted genes in KR01 used as the background for the statistical analysis. The Fisher's Exact test statistic and the ‘elimination’ algorithm were applied to correct for the hierarchical structure of GO terms. For subcellular localization prediction, signal peptides in the protein sequences were predicted using MITOPROT II (Claros & Vincens, 1996), iPSORT (Bannai et al., 2002), and DeepLoc‐1.0 (Almagro Armenteros et al., 2017), TargetP – 2.0 (Emanuelsson et al., 2007), TPpred2 (Savojardo et al., 2014), and CELLO v.2.5 (Yu et al., 2014). The previously generated chromatophore‐targeting peptide (crTP) protein database (Lhee et al., 2021) was used to identify putative biochemical CCM‐related proteins with a crTP.

### Phylogenetic analysis of biochemical CCM‐related genes in KR01

A taxonomically diverse database was created so phylogenetic analysis could be conducted with the biochemical CCM‐related genes identified in KR01 to assess their origin in *Paulinella*. Proteins from NCBI RefSeq, plus available algal and protist genome and transcriptome data from dbEST, TBestDB, the JGI Genome Portal (https://genome.jgi.doe.gov) and the Moore Microbial Eukaryote Transcriptome Sequencing Project (MMETSP) (Keeling et al., 2014) were collected and partitioned into four sets based on taxonomic origin. These sets were composed of sequences from (i) bacteria, (ii) Opisthokonta, (iii) the remaining sequences not from bacteria or Opisthokonta, and (iv) sequences from the MMETSP database. The identified KR01 proteins were searched independently (BLASTp v1.10.1; ‘‐max_target_seqs 2000‐evalue 1000’) against each of the four (i–iv) database subsets. For each query, the top hits against each set were filtered (*e*‐value ≤1e^−10^), combined, and sorted by bitscore (descending order), with a taxonomically broad selection of top hits extracted from the resulting sorted list. Alignments were generated using MAFFT (v7.453; ‘‐‐localpair ‐‐maxiterate 1000’) (Katoh & Standley, 2013) and trimmed for poorly aligned regions using trimAl (v1.4.1; ‘‐automated1’) (Capella‐Gutierrez et al., 2009) for each of the target proteins and their selected top hits. The selected top hits from KR01 biochemical CCM‐related proteins annotated as the same enzyme were combined, duplicate hit sequences were removed, aligned together with their corresponding KR01 protein sequences using MAFFT (v7.453; ‘‐‐localpair ‐‐maxiterate 1000’), and trimmed for poorly aligned regions using trimAl (v1.4.1; ‘‐automated1’). IQTREE (v1.6.12; ‘‐m MFP ‐bb 2000 ‐nm 2000 ‐quiet’) (Nguyen et al., 2015) was used to construct a maximum‐likelihood phylogenetic tree from each of the alignments, with automatic model selection (Kalyaanamoorthy et al., 2017) and node support tested via 2000 ultrafast phylogenetic bootstraps (Minh et al., 2013). Trees and alignments were visualized together using TreeViewer (v1.2.2; https://github.com/arklumpus/TreeViewer).

## RESULTS

### Novel genes comprising a putative CO_2_ delivery system in KR01

**FIGURE 1:**
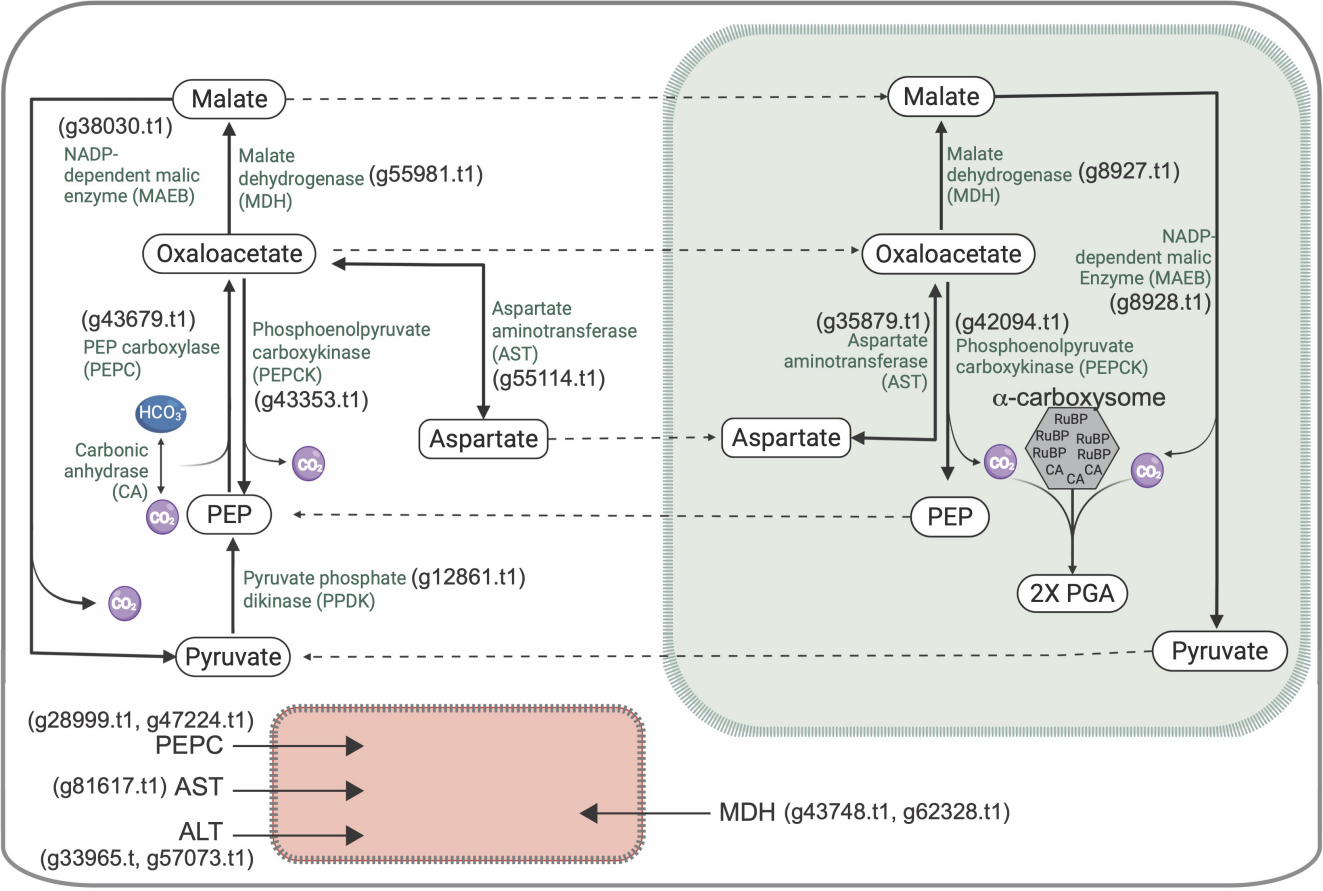
Working model of the putative CO_2_ delivery system to the chromatophore localized α‐carboxysome in photosynthetic *Paulinella* (KR01). The major processes predicted to occur in the cytoplasm and mitochondria (red box with dashed outline) and the chromatophore (green box) are shown, as are the numbers for each relevant KR01 gene. Dashed arrows represent the predicted direction of transport of metabolites between the cytoplasm and the chromatophore. The α‐carboxysome (based on Roberts et al., 2012; Ni et al., 2022) contains multiple RuBisCOs (RuBP) and carbonic anhydrases (CA, for CsoSCA). Created with BioRender.com.

**FIGURE 2:**
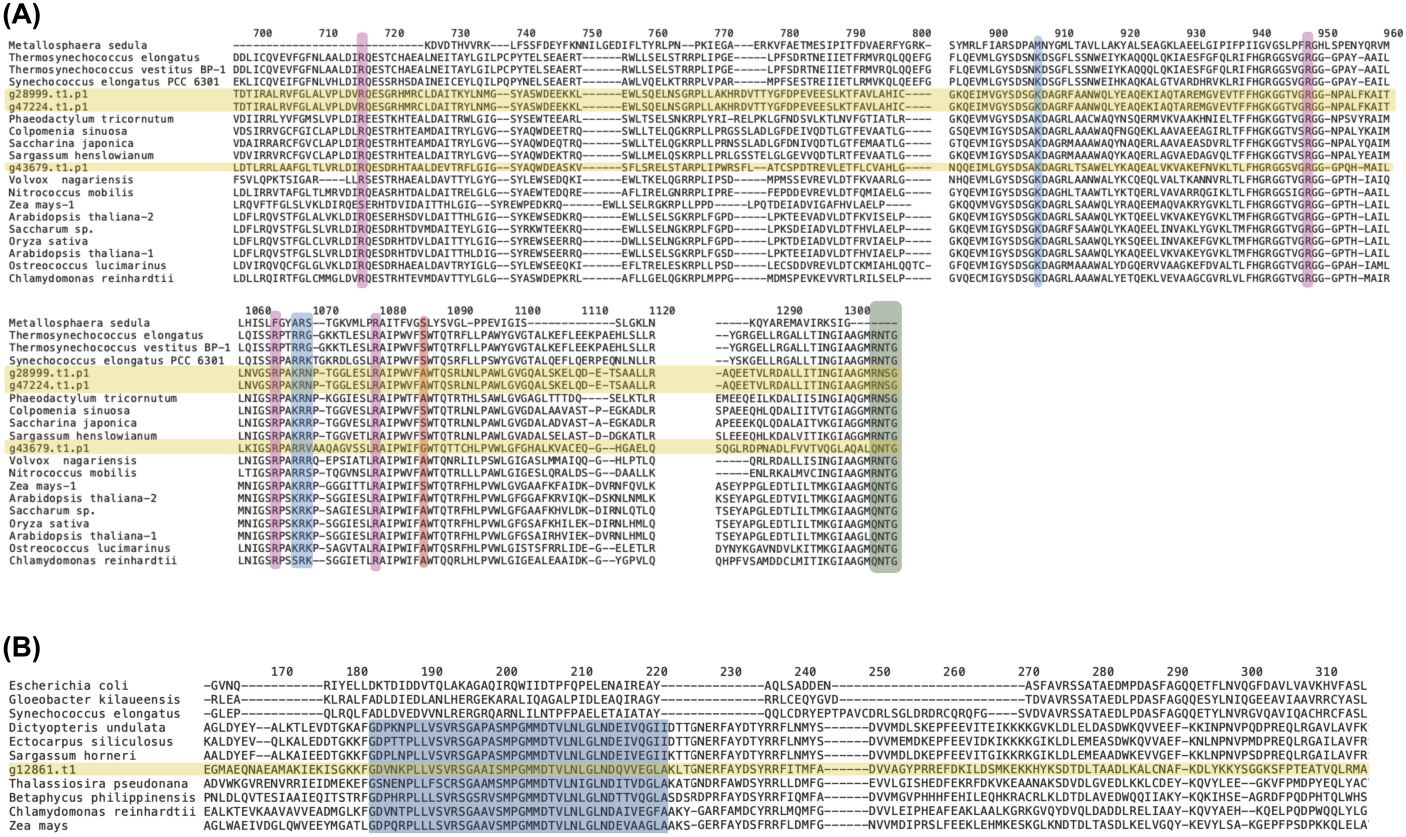
Alignments of PEPC (A) and PPDK (B) proteins from KR01 (in the yellow field) with those in other organisms. (A) The C‐terminal end of the PEPC protein alignment is shown with the conserved PEP binding sites (pink), bicarbonate binding site (blue), C‐terminal tetrapeptide (green), and the amino acid residues corresponding to position 774 in plant PEPC proteins (red) highlighted. (B) A section of the PPDK protein alignment is shown with the identified sequence signature for the real PPDK enzyme highlighted in blue. Sequences were aligned using Clustal Omega 1.2.3 in Geneious Prime 2022.1.1 and the colour annotations were created with BioRender.com. Numbering indicates the position of residues within the alignment.

Our analysis of KEGG pathways in the KR01 predicted proteome (Lhee et al., 2021) suggested the existence of a putative biochemical CCM based on several candidate genes of interest. Three of these genes encode phosphoenolpyruvate carboxylase (PEPC, EC 4.1.1.31), two genes encode phosphoenolpyruvate carboxykinase (PEPCK, EC 4.1.1.49), and one gene encodes a pyruvate orthophosphate dikinase (PPDK, EC 2.7.9.1) (Table 1). Of the three genes that encode PEPC, two have identical protein sequences and are predicted to be mitochondrion‐targeted (Figure 1; Supplemental Table 2; genes g28999.t1.p1 and g47224.t1.p1). Examination of the KR01 PEPC protein sequences shows that each contains a PEP (Figure 2A, pink highlight) and bicarbonate (HCO_3_^−^) (Figure 2A, blue highlight) binding site (Kai et al., 2003), which are required for catalytic function. One of the PEPC proteins, with uncertain (but likely cytosolic) localization (g43679.t1.p1; Supplemental Table 2), contains a QNTG C‐terminal tetrapeptide which is typical of plant‐type PEPC enzymes (PTPC), whereas the other two contain an RNSG C‐terminal tetrapeptide which is present in bacterial (mitochondrion)‐type PEPC (BTPC) (Chi et al., 2014; O'Leary et al., 2011). None of the KR01 PEPC proteins contains a crTP (Figure 1).

**TABLE 1:** Putative biochemical CCM‐related gene modules in KR01.

C_4_‐cycle module	Appr.	Name	EC.	KEGG	Putative localization	KR01 protein ID	Log_2_fold change			
							BC	HL	HLBC	pH
**Carboxylation**	**PEPC**	**Phosphoenolpyruvate carboxylase**	**4.1.1.31**	**K01595**	**Mitochondrion**	**g28999.t1**	**−0.884451073**	**1.239062409**	**−0.745684994**	**−1.413876134**
					Mitochondrion	g47224.t1	−2.296565925	0.443130519	−1.973318812	−1.966337213
					Cytosolic	g43679.t1	−1.449794973	−0.86792766	−2.039291322	−1.465061624
	α‐CA	Alpha carbonic anhydrase	4.2.1.1	K01672		g18485.t1	−0.449446165	0.472296912	−1.763779853	−1.601067633
						g61179.t1	0.246680485	3.758383137	1.33977238	−1.527304767
						g3883.t1	−3.680345858	0.187962784	−5.282759222	−4.50240065
				K18245		g77398.t1	−3.237350688	1.059681749	−5.651768406	−4.253142808
						g77400.t1	−3.885604937	0.265053401	−5.766198723	−4.5643956
						g64672.t1	0.062777578	−0.438683799	0.50710676	1.259061445
	β‐CA	Beta carbonic anhydrase		K01673		g83283.t1	−2.146605778	1.068256092	−2.236860762	−1.899504082
						g51312.t1	−1.585630853	1.324747101	−2.555995587	−0.986776479
						g79711.t1	−1.061896567	−2.28088176	−1.759875535	0.023510981
						g79490.t1	−1.534561714	−3.100965006	−1.741036944	−3.347851199
	γ‐CA	Gamma carbonic anhydrase		K01743		g60394.t1	−0.362335792	−0.321484374	−0.097668914	0.560930924
**Transfer molecule generation**	**AST**	**Aspartate aminotransferase**	**2.6.1.1**	**K14454**	Cytosolic	g55114.t1	−0.539925648	0.384138	−0.064597474	−0.109791888
					Chromatophore	g35879.t1	0.184503106	1.580914053	0.386776012	−0.977680264
				**K14455**	Mitochondrion	g81617.t1	0.124931903	0.803526037	0.357019807	−0.437170163
	**MDH**	**Malate dehydrogenase**	**1.1.1.37**	**K00024**	Chromatophore	g8927.t1	−0.29916225	1.706748761	0.583566697	−1.657175649
					Cytosolic	g55981.t1	−0.164806257	1.391692703	0.487790501	−0.374729187
				**K00026**	Mitochondrion	g62328.t1	−0.52767045	0.565619136	−0.053237543	−0.796394182
					Mitochondrion	g43748.t1	0.564264914	0.12021415	0.350028083	−0.472759283
	**ALT**	**Alanine aminotransferase**	**2.6.1.2**	**K00814**	Mitochondrion	g33965.t1	−0.469003421	0.165907877	−0.52329943	−1.342726235
					Mitochondrion	g57073.t1	−0.439717165	−1.146340526	−0.328825021	−1.114855113
**Decarboxylation**	**PEPCK**	**Phosphoenolpyruvate carboxykinase**	**4.1.1.49**	**K01610**	Cytosolic	g43353.t1	0.285547378	1.158581123	0.505789895	−1.201148243
					Chromatophore	g42094.t1	−0.153373714	0.560775628	−0.635379085	0.240328334
	**MAEB**	**NADP‐dependent malic enzyme**	**1.1.1.40**	**K00029**	Cytosolic	g38030.t1	0.026033663	0.04135517	0.25801251	0.633869426
					Chromatophore	g8928.t1	−0.135115343	0.996543959	−0.565549845	0.465174824
**Regeneration of PEP**	**PPDK**	**Pyruvate orthophosphate dikinase**	**2.7.9.1**	**K01006**	Cytosolic	g12861.t1	0.482798144	0.815942603	0.102799242	−0.726744773
	AMK	Adenylate kinase	2.7.4.3	K00939		g25648.t1	0.633111751	1.159659459	0.224850507	−0.286497563
						g1121.t1	0.229618317	0.461044608	0.228014713	−0.281962936
						g39743.t1	0.131687817	1.290120751	−0.023272722	−0.609559291
						g82166.t1	−0.275671455	0.273865257	−0.046075398	0.383276444
						g10172.t1	−0.325087898	0.118198226	0.122658836	−0.047347838
	PPase	Pyrophosphate phosphohydrolase	3.6.1.1	K01507		g46682.t1	−0.236636562	0.344399574	0.26517787	−0.2572601

*Note*: List of central core genes (bold with green highlight) and other closely related genes and their expression under different treatments. The log_2_fold change values that represent significantly (adjusted *p*‐value <0.05) up‐regulated (orange) or down‐regulated (blue) genes are highlighted for each treatment. The predicted localization (Loc) of the proteins is shown (C, cytosolic; CR, chromatophore; M, mitochondrion).

Only one copy of pyruvate orthophosphate dikinase (PPDK, EC 2.7.9.1) is encoded in the KR01 nuclear genome (Table 1; g12861.t1). This PPDK possesses neither a signal sequence nor a transit peptide and is most likely localized in the cytosol (Supplemental Table 2). Two copies of PEPCK were identified in KR01 (Table 1), one of which contains a crTP (g420994.t1) (Lhee et al., 2021; Singer et al., 2017) and is likely targeted to the chromatophore. In addition to encoding a PEPCK decarboxylation enzyme, the KR01 nuclear genome contains two genes that encode NADP‐malic enzyme (MAEB, EC 1.1.1.40), one of which contains a crTP (g8928.t1), whereas the other is likely cytosolic (g38030.t1).

Four copies of the malate dehydrogenase (MDH) gene were identified in KR01, with two of the encoded proteins predicted to be in the mitochondrion (g62328.t1, g43748.t1), one in the chromatophore (g8927.t1), and one in the cytosol (g55981.t1) (Table 1, Supplemental Table 2). Three gene copies of aspartate aminotransferase (AST, EC 2.6.1.1), which catalyses the formation of OAA from aspartate, were identified in KR01, with one encoded protein likely targeted to the chromatophore (g35879.t1) and the others to the cytoplasm (g55114.t1) and mitochondria (g81617.t1). Finally, there were two gene copies of alanine aminotransferase (ALT, EC 2.6.1.2), with the encoded proteins predicted to be localized to the mitochondrion (Table 1, Supplemental Table 2). Phylogenetic analysis shows that all the CCM‐related genes examined in KR01, except MDH g8927.t1, are of eukaryotic origin (i.e., not the result of recent HGT provenance or EGT) (Supplemental Dataset 1). There are single to multiple copies of many of the KR01 CCM‐related genes in the *P. chromatophora* proteome, that was predicted using a cDNA assembly (Nowack et al., 2016; see Supplemental Dataset 1). These data suggest that the evolutionary trajectories of KR01 and *P. chromatophora* have diverged significantly since their split (Bhattacharya et al., 2023). Given the lack of a high‐quality genome assembly for *P. chromatophora* and no experimental data regarding a putative CCM in this species, we have based the conclusions of the present study solely on KR01. A detailed analysis of *P. chromatophora* may turn up additional, novel insights into pathways of CCM evolution in the *Paulinella* lineage.

### Model for the putative CO_2_ delivery system in KR01

Based solely on the number, functional annotation, and putative subcellular localization of the predicted proteins, we developed a hypothetical model for CO_2_ delivery to the chromatophore in KR01 that includes components of a biochemical CCM (Figure 1). This model posits that the PEPC carboxylation reaction is localized in the cytosol/mitochondria, suggesting that OAA is generated in the cytosol before it is transaminated to aspartate by AST or reduced to malate via MDH, with both of these reactions occurring in the cytoplasm or the chromatophore, although there are mitochondrion targeted versions of these enzymes in KR01. Decarboxylation of malate and OAA in the cytosol is possible because of cytosol‐localized MAEB and PEPCK activities, which would convert these molecules to pyruvate and PEP (respectively) plus CO_2_. Transfer molecules, in the form of OAA, malate, or aspartate, may enter the chromatophore (mechanism currently unknown) where decarboxylation via the chromatophore targeted PEPCK and MAEB enzymes would release CO_2_ in the compartment containing RuBisCO (Figure 1). Finally, the regeneration of PEP from pyruvate by PPDK likely occurs only in the cytosol.

### Quantitative and qualitative transcriptome information

To study the expression patterns of genes related to the putative biochemical CCM proposed here, we generated RNA‐seq data from cells exposed to one of four treatments for 6 h: (1) HL (HL; *n* = 4), used to induce or upregulate the CO_2_ delivery system (150 μmol photons m^−2^ s^−1^, pH = 6.8, [CO_2aq_] = 14 μM); (2) bicarbonate (HCO_3_^−^) supplementation (BC; *n* = 4) under low light, which provided a nearly 8‐fold increase in the extracellular concentration of CO_2_ (pH = 8.0, [CO_2aq_] = 110 μM) relative to our standard batch culture and the HL treatment, and is therefore expected to suppress the synthesis of enzymes involved in a putative biochemical CCM; (3) combined HL and bicarbonate treatment (HLBC, pH = 8.0, [CO_2aq_] = 110 μM, *n* = 4) to determine if the putative CCM enzymes are downregulated in response to increased CO_2_ regardless of light intensity; and (4) low CO_2_/pH 8.0 treatment ([CO_2aq_] = 14 μM, pH = 8.0, *n* = 4), because the addition of HCO_3_^−^ to cultures resulted in a pH shift from 6.8 to 8.0; this treatment was included to control for any effects of pH on gene expression or biochemical CCM function, independent of a change in external CO_2_. All treatments were compared to samples from a low light (LL), low CO_2_ control treatment (Ctrl., 15–20 μmol photons m^−2^ s^−1^, [CO_2aq_] = 14 μM, pH = 6.8, *n* = 4) (see Materials and Methods). Note that no differences in growth rate were observed among the four treatments or the control over the 6 h incubations used in this study of gene expression. In total, 1,301,208,566 high‐quality paired‐end reads were retained across all samples after adapter and quality trimming (average of 65 million reads per sample). Of these, 1,060,767,326 (81.52%) were mapped to the KR01 reference genome (Lhee et al., 2021). The “unique exon count” value returned for each gene by the CLC Genomics Workbench read mapping analysis tool was by DESeq2 Version 1.34.0 (Love et al., 2014) to identify differentially expressed genes (DEGs) (Supplemental Tables 1 and 3).

Sample expression profiles were compared using principal component analysis (PCA), with pairwise correlation values constructed using DESeq2 normalized counts. This analysis allowed us to visualize the similarity between each of the samples and to identify any outliers. In general, samples from the same treatment were grouped together in the PCA and distinct from samples from other treatments (Supplemental Figure 1). The BC and HLBC treated samples clustered together when compared to other treatments. This may represent a genuine biological signal (i.e., high similarity in gene expression profiles of samples from the treatments) (Supplemental Figure 1A). Based on their position in the PCA plot, one outlier sample from each treatment group (samples: control1, pH 1, HL3, and BC4) was detected; this effect is also obvious from the results of the pairwise correlation analysis shown in Supplemental Figure 1B. After close inspection of the treatment protocol, sample collection, and RNA extraction protocol and results, it remains unclear which factors led to these samples having expression profiles that are distinct from all other samples exposed to the same treatment. To eliminate biases or excess noise in the analysis, these samples were classified as outliers and removed from the analysis. This resulted in a total of 851,155,573 mapped reads that were subsequently used in the downstream analyses.

### Differential gene expression analysis

DEGs from all treatment groups were identified using the DESeq2 package in RStudio 2021.9.2.382 (Team, 2022). In total, 23,995 genes were expressed in the HL plus control samples, of which 10,432 were significantly (adjusted *p‐*value <0.05) differentially expressed between the two treatments; 5120 of these genes were upregulated and 5312 were downregulated in HL. There were 24,621 genes expressed in the BC plus Control samples, of which 4371 were significantly differentially expressed; 1830 were upregulated and 2541 were downregulated in BC. There were 24,899 genes expressed in the HLBC plus control treatment samples, of which only 3899 were significantly differentially expressed; 1393 were upregulated and 2506 were downregulated in HLBC. Finally, there were 24,584 genes expressed in the low CO_2_/pH 8 plus Control treatment samples, of which 8609 were significantly differentially expressed; 4212 were upregulated and 4397 were downregulated in the low CO_2_/pH 8 sample. A total of 1257 significant DEGs were found to be associated with all treatments. A similar number of unique DEGs (i.e., not differentially expressed in other treatments) were identified in the BC and HLBC treatments (374 and 324, respectively), whereas the HL treatment had the largest number of unique DEGs (3955) (Supplemental Figure 2; the overlap in DEGs between treatments, is shown in this figure).

### Functional annotation of DEGs

Genes differentially expressed in each treatment were functionally annotated using BLASTp and KAAS (Supplemental Tables 4–7). To understand more about the functions of the DEGs in each treatment, gene ontology (GO) terms were assigned to the predicted proteins using PANNZER2 (Toronen et al., 2018). Significant DEGs (adjusted *p‐*value <0.05) from each treatment were analysed for enrichment of GO terms using the topGO R package (Alexa & Rahnenführer, 2010), using all genes predicted in KR01 as the background; Fisher's exact test statistic and the ‘elimination’ algorithm were applied to correct for the hierarchical structure of GO terms. The top 30 GO terms from each category (biological processes, cellular component, and molecular function) for each treatment, ordered by Fisher's exact test *p*‐values, are shown in Supplemental Figure 3. Some of the top, significantly enriched biological processes include “translation”, “proton transmembrane transport”, “protein folding”, “glycolytic process”, “acyl‐CoA biosynthetic process”, “cellular protein‐containing complex”, “glycine metabolic process”, “DNA replication”, “tricarboxylic acid cycle”, “DNA duplex unwinding”, “sulfur compound biosynthetic process”, “heterochromatin assembly”, “cellular response to unfolded protein”, and “carbohydrate derivative biosynthesis” (Supplemental Figure 3). The full GO term enrichment results for each treatment are presented in Supplemental Tables 8–11. The majority of KR01 proteins associated with expressed genes have unknown functions. Of the top 20 DEGs with the highest absolute fold‐change in each treatment, only one gene in the HL, one gene in the BC, five genes in the HLBC, and one gene in the pH treatment had KO numbers assigned, or top hits to proteins in the NCBI NR database that had unambiguous descriptions (i.e., not “unknown” or “hypothetical” function; Supplemental Tables 4–7). That is, the majority of the top 20 DEGs had unknown functions. Of the top 20 DEGs in each treatment, 10 were shared between HL and pH, two between BC and HLBC, and two between HLBC and pH (14 total, all downregulated).

### Differential expression of putative biochemical CCM‐related genes

**FIGURE 3:**
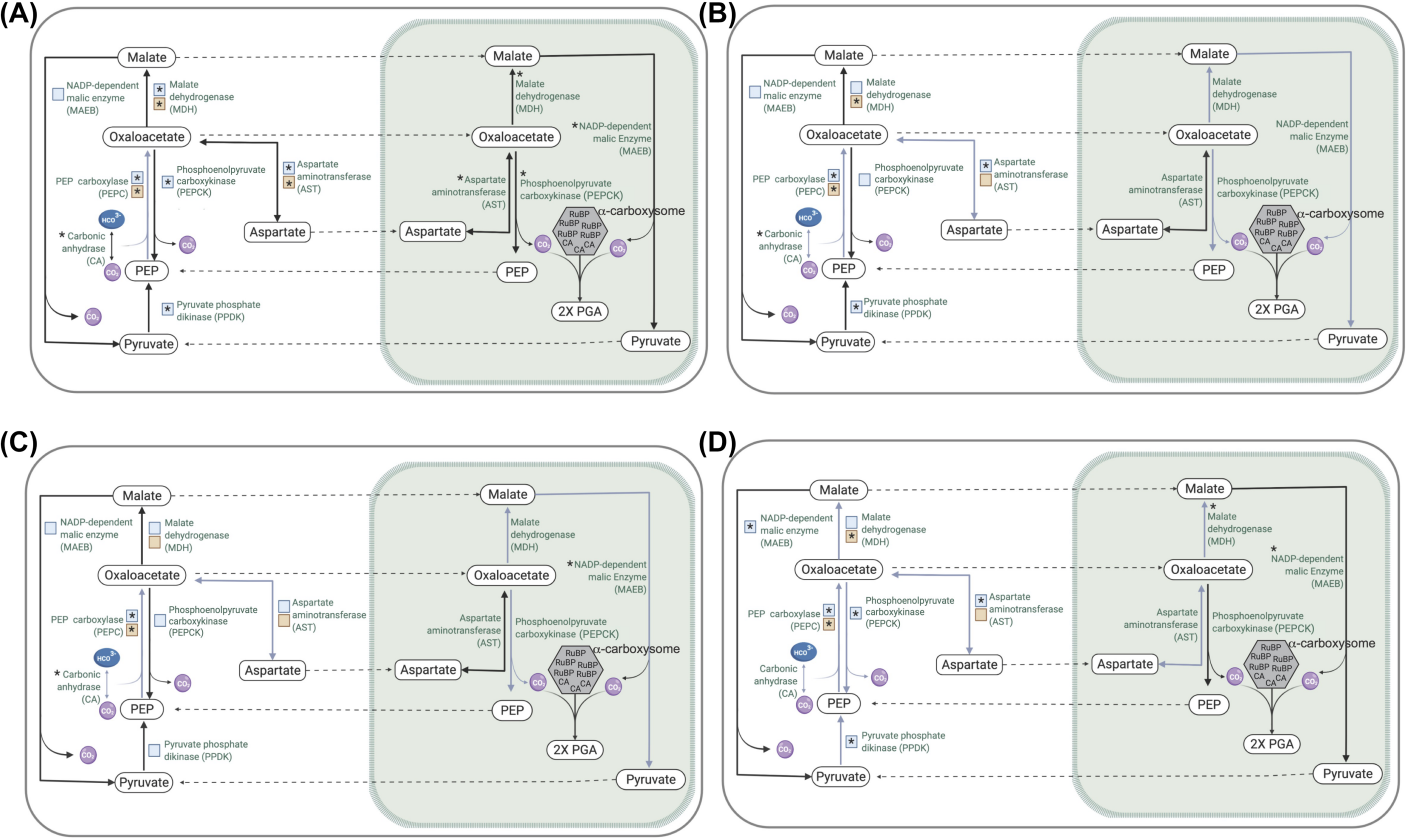
Working model of the putative CO_2_ delivery system to the chromatophore localized α‐carboxysome in *Paulinella* (based on Figure 1), showing the results of differential gene expression analysis. Expression pattern of CCM‐related genes in (A) high light (HL; induced condition), (B) added bicarbonate (BC; inhibition condition) under low light, (C) double treatment (HLBC), and (D) under low light, pH shifted from 6.8 to 8 (pH) treatments. Solid black arrows indicate upregulation of expression in response to a treatment. Solid grey arrows indicate the downregulation of expression in response to treatment. An asterisk indicates significance (adjusted *p‐*value <0.05) up‐ or downregulated in response to treatment. Enzymes encoded by multiple gene copies are represented by coloured squares, with copies localized to the cytoplasm represented by blue squares and copies localized to the mitochondria by orange squares.

Differential expression analysis was done to determine if and how the putative CCM‐related genes in KR01 respond to environmental change. The genes were categorized into different modules based on their functions (Table 1). PPDK, the main enzyme involved in PEP regeneration was significantly upregulated under HL. However, PPDK was also upregulated in the BC and HLBC treatments (Table 1, Figure 3), but was downregulated in response to elevated pH. The upregulation of PPDK at elevated CO_2_ is inconsistent with a role for this enzyme in a CO_2_ delivery system in *Paulinella*. In this case, an alternative source of PEP (see PEPCK, below) would be needed to provide a carboxylation substrate for PEPC.

The carboxylation of PEP is catalysed by PEPC using HCO_3_^−^. The two putatively mitochondrion‐targeted PEPC proteins were significantly upregulated under HL and significantly downregulated under the BC and HLBC treatments (Table 1, Figure 3), consistent with their role in a CO_2_ delivery system. They were also significantly downregulated in the low CO_2_/pH 8.0 treatment. As described above, the rapid conversion of CO_2_ to the HCO_3_^−^ used in the carboxylation reaction is facilitated by CA. Eleven CA genes encoding proteins that lack a crTP (and are therefore unlikely to be chromatophore targeted) were identified in the genome of KR01. Six of the genes are α‐CA, four are β‐CA, and one is a γ‐CA. The three containing a putative mitochondrial targeting peptide are two β‐CAs (g83283.t1, g51312.t1) and one γ‐CA (g60394.t1). Six of these CA genes were significantly upregulated in response to HL with the α‐carbonic anhydrase gene, g61179.t1, showing the highest log_2_fold change (3.76). Five of these six CA genes were significantly downregulated under the BC, HLBC, and low CO_2_/pH 8.0 treatments (Table 1, Figure 3). The latter result is not consistent with a CCM. Therefore, specific roles and localization of these CAs merit further research.

In KR01, PEPCK and NADP‐ME which perform decarboxylation reactions are each encoded by two genes; one PEPCK and one NADP‐ME protein is localized to the chromatophore whereas the other PEPCK and NADP‐ME are likely cytosolic (Table 1, Figure 2B). Under HL, both copies of PEPCK were significantly upregulated. However, under BC, the cytosolic copy of PEPCK was upregulated, whereas the crTP‐containing PEPCK copy was downregulated (neither was significantly differentially regulated). A similar pattern was observed for NADP‐ME with the cytosol localized copy upregulated under both conditions (HL and BC; although not significantly upregulated), whereas the chromatophore targeted copy was significantly upregulated under HL and non‐significantly downregulated under BC, as expected if the chromatophore targeted NADP‐ME played a role in a biochemical CCM (Table 1, Figure 3). One or both chromatophore‐targeted copies of these decarboxylases could produce CO_2_ near RuBisCO, with PEPCK also regenerating PEP.

The delivery of CO_2_ to the chromatophore would require carrier molecules such as OAA, malate, or aspartate to transfer carbon from the cytosol to the site of RuBisCO. The short‐lived OAA molecules produced by PEPC are quickly converted into malate or aspartate by MDH and AST (both of which are encoded on the KR01 genome), before being transported to the site of decarboxylation. There are three genes encoding AST, one of which contains a crTP, a second is putatively mitochondrion targeted, and the third is cytosolic (Figure 1). Two of the AST genes, including the one encoding a crTP (Table 1), were significantly upregulated under HL and showed non‐significant upregulation under BC and HLBC treatments. The third, putative mitochondrion‐targeted protein (g81617.t1) was significantly upregulated under HL but downregulated under BC (significantly), HLBC (non‐significantly), and low CO_2_/pH 8 treatments (non‐significantly). Four copies of the MDH enzyme were identified in KR01. One is chromatophore targeted (g8927.t1), one is cytosolic (g55981.t1), and two are mitochondrion targeted (g62328.t1, g43748.t1). Genes encoding all but one mitochondrion‐targeted protein (g43748.t1) were significantly upregulated under the HL treatment, with the cytosolic copy showing log_2_fold up‐regulation of 1.4. However, the expression of these three up‐regulated genes under BC and HLBC treatments varied, although they generally showed weak (non‐significant) up or downregulation (Table 1, Figure 3). Two genes encoding alanine aminotransferase (ALT) exist in the KR01 genome with the encoded proteins putatively mitochondrion targeted (Table 1). One gene (g57073.t1) appears to be non‐significantly downregulated under all conditions, whereas the other (g33965.t1) is significantly upregulated under HL and significantly downregulated under all other treatments. Based on these results, it appears that one or more AST, MDH, and ALT genes have transcription patterns in *Paulinella* consistent with a role in delivering CO_2_ to the chromatophore.

### Photorespiratory genes

The C_2_ photorespiratory cycle converts the two‐carbon product of RuBisCO oxygenation, 2‐P‐glycolate (2‐PG), into glycine and ultimately serine and CO_2_. The decarboxylation of glycine is catalysed by the multi‐enzyme glycine decarboxylase complex (GDC). We identified genes for critical proteins of the GDC, including two aminomethyl‐transferring glycine dehydrogenases (g40133.t1, g40134.t1) and a glycine cleavage system protein H (g44532.t1) in the KR01 transcriptome data. The expression of both glycine dehydrogenases was not significantly affected by a shift to HL but was significantly downregulated in the high CO_2_ treatments (BC and HLBC) and in the low CO_2_/pH 8 treatment. The glycine cleavage system protein H was downregulated under all treatments relative to the low light, low CO_2_ and low pH control. These expression patterns are not consistent with a C_2_ photorespiratory cycle that increases with oxygen production at high irradiance, but rather, suggest that glycine decarboxylation is upregulated by low external pH. In contrast, alanine‐glyoxylate aminotransferase, the enzyme that catalyses the production of glycine from glyoxylate just upstream of the GDC in the C_2_ photorespiratory pathway, was upregulated under high light and downregulated under high light plus high CO_2_ (HLBC) and low CO_2_/pH 8. This suggests that under high light, a bottleneck may occur at the glycine decarboxylation step leading to a buildup of glycine and inefficient regeneration of 3‐PGA for the Calvin‐Benson cycle. The KR01 gene expression data lacked all enzymes of the alternative glycerate and decarboxylation photorespiratory pathways (Eisenhut et al., 2008). A gene for a mitochondrial glycine transporter‐like protein (g6365.t1) was significantly upregulated under HL but was not significantly affected by HL plus high CO_2_ or low CO_2_/pH 8. However, the expression of an alanine glycine permease (g2033.t1) was not significantly regulated by high light or high light plus high CO_2_ but was strongly downregulated by low CO_2_/pH 8. Note that both these glycine transporters are not present in the BC transcriptomes. Overall, the expression of C_2_ cycle genes indicates that *Paulinella* lacks or has a C_2_ cycle that is uncoordinated with the production of 2‐PG produced by RuBisCO oxygenase activity.

### Phylogeny of CCM‐related proteins in KR01

Phylogenetic analysis of the CCM‐related genes was performed to determine whether they are of eukaryotic (host) provenance or originated via HGT or EGT. All these predicted proteins in KR01 are of eukaryotic origin except for the crTP‐containing copy of MDH (g8927.t1; Supplemental Dataset 1). This MDH gene is positioned in a well‐supported clade (bootstrap support 100%) with sequences from predominantly alpha‐proteobacteria. It should be noted that the MDH and PCK combined gene‐set trees, as well as the g8927.t1, g55981.t1, and g8928.t1 gene trees did not converge after 2000 iterations. Because the placement of the KR01 genes was congruent across these trees, their topologies were considered robust (for all trees, see Supplemental Datasets 1 and 2).

## DISCUSSION

There has been much interest in developing *Paulinella* as a model system for the analysis of organellogenesis because it is the only known intermediate stage of plastid primary endosymbiosis and has many features associated with “evolution in action” (Calatrava et al., 2022; Gabr et al., 2020; Stephens et al., 2021; Van Etten et al., 2023). Currently, knowledge of its photosynthetic and CO_2_ fixation pathways is limited, and we still do not fully understand the mechanisms associated with the adaptation of this species to its photosynthetic organelle. These issues are exacerbated by the extremely slow growth rate of *Paulinella* cells in culture that requires months of effort to generate sufficient biomass for omics or other analyses. In this study, we find that the KR01 chromatophore genome does not encode a bicarbonate transporter, as would be expected for this cyanobacterial‐derived organelle. In contrast, analysis of the chromatophore genome from *P. chromatophora* (Nowack et al., 2008; NCBI accession NC_011087) and *P. longichromatophora* (Lhee et al., 2019; NCBI accession MG264610) reveal the presence of an inorganic carbon transporter gene (*ictB*). This gene is absent from the KR01 chromatophore genome (Lhee et al., 2017; NCBI accession KX897545), although the flanking genes in this region are conserved in *P. chromatophora* and *P. longichromatophora* (upstream: phosphotransferase superclass; downstream, tRNA (guanine‐N(7)‐)‐methyltransferase), indicating a deletion event in KR01. Homologues of *ictB* could not be identified in the nuclear gene inventory of KR01, suggesting the gene has been lost from this lineage. Phylogenetic analysis of IctB demonstrates that the highly diverged chromatophore encoded proteins (potentially indicating weakened selection) are of α‐cyanobacterial origin in *P. chromatophora* and *P. longichromatophora* (Supplemental Figure 4). Knockdown of *ictB* demonstrated it as being necessary for HCO_3_^−^ transport in *Synechococcus* sp. PCC 7942 (Bonfil et al., 1998). Given this result, we propose that the CCM is, as for many other features associated with photosynthesis (see above), in a transitional phase in *Paulinella* species, with bicarbonate transport in *P. chromatophora* and *P. longichromatophora* likely able to support the native α‐carboxysome in these taxa. It is possible that the biochemical CCM we propose to exist in KR01 may be the outcome of the loss of *ictB* (see below), although this is currently speculation. Perhaps most intriguing, different CCMs may be evolving in KR01 and other *Paulinella* species. Given this knowledge, we report that the KR01 nuclear genome encodes genes that may improve the delivery of CO_2_ to the chromatophore via a putative biochemical CCM to enhance the function of the α‐carboxysome in this species. The upregulated expression of target genes under HL, and downregulation when experiencing supplementation with bicarbonate (either in LL or HL) are consistent with this hypothesis.

### DEG analysis in KR01

Analysis of the expression patterns of KR01 genes involved in the putative biochemical CCM shows that under HL when we would expect higher RuBisCO activity and a higher demand for CO_2_ at the site of carbon fixation, many of these genes are significantly upregulated (adjusted *p‐*value <0.05; Table 1, Figure 3). With few exceptions, the opposite is true under BC; the genes are (often significantly) downregulated when bicarbonate is added to the culture medium which increases CO_2_ availability and may reduce the need for CCM activity (Table 1). Moreover, genes upregulated under HL are suppressed (i.e., downregulated or not significantly upregulated) under HL with the addition of bicarbonate (the HLBC treatment). This suggests that many of the CO_2_ delivery genes in KR01 are responding to the external CO_2_ concentration and not directly to light intensity (at the higher light intensity more CO_2_ would be fixed, reducing the intracellular CO_2_ concentration). Some of these genes also respond to the pH treatment in which the bicarbonate concentration was elevated with respect to the control, suggesting that, as in plants, HCO_3_^−^ might be used as a pH indicator in photosynthetic *Paulinella* and some of the genes may be responding to changes in pH as well as CO_2_. We also found that, whereas the HL and pH treatments shared the greatest number of significant DEGs, they also had the most unique, significant DEGs (3955 and 2419, respectively). This suggests that HL and pH affect multiple pathways, as expected, and elicit a broad transcriptional response by the cell. In contrast, the BC treatment resulted in only 374 unique DEGs (Supplemental Figure 2). The top 20 genes with the largest absolute fold change in each treatment have unknown functions (Supplemental Tables 4–7). This suggests that in KR01 (and potentially in *Paulinella* more generally), ‘dark’ genes of unknown function (e.g., regulatory roles; Stephens et al., 2018) may be of high importance in the stress response.

In contrast to genes associated with the putative biochemical CCM, genes encoding critical components of a putative, ancestral KR01 biophysical CCM did not follow the expected transcription pattern of up‐regulation under HL and down‐regulation in high CO_2_. For example, expression of the single nuclear gene that encodes an α‐carboxysome component, *csoS4A*, did not change markedly under either HL (log_2_fold change = 0.155; adjusted *p*‐value = 0.872) or high CO_2_ (log_2_fold change = −0.269; adjusted *p*‐value = 0.842) when compared to the low light/low CO_2_ control. The other treatments (HLBC, pH) also did not result in significant expression differences for this gene. In addition to carboxysome proteins, biophysical CCMs in cyanobacteria depend on bicarbonate transport (Price et al., 2008). Three putative eukaryotic bicarbonate transporters from the solute carrier (SLC) 26 family and none from the SLC4 family were identified in KR01. One *SLC26* gene (g61426.t1) was upregulated in both HL and BC treatments, inconsistent with the expected pattern of a CCM‐critical gene, whereas the other two were downregulated by HL and did not significantly change in the other treatments (Supplemental Tables 4–7). The putative biochemical CCM in KR01 may therefore support or compensate for an ineffective or poorly regulated biophysical CCM, perhaps due to the loss of the putative cyanobacterial bicarbonate transporter *ictB*. In that case, the biochemical CCM may deliver inorganic carbon to the chromatophore where a combination of elevated pH and carbonic anhydrase trap it as HCO_3_^−^, which is subsequently converted to CO_2_ and then fixed by carboxysome‐encapsulated carbonic anhydrase and RuBisCO, respectively. Such a scenario could be supported by one or more of the nuclear‐encoded CAs that were significantly upregulated in response to HL and downregulated under elevated CO_2_, but the localization of these nuclear‐encoded CAs and how they may be translocated to the chromatophore is currently unclear. Given that photosynthesis‐related genes on the chromatophore genome (e.g., *psbA*, *psaA*) appear to lack light regulation (Zhang et al., 2017), the *csoS4A* result suggests that nuclear control of α‐carboxysome activity may also not be light‐regulated. This hypothesis extends to the shell carbonic anhydrase, *csoSCA*, that is key to biophysical CCM function. Nonetheless, the relocation of a key structural component of the α‐carboxysome to the nuclear genome may allow the amoeba to control the catalytic activity of this microcompartment (Cai et al., 2009). Such control is consistent with the analysis of nucleotide biosynthesis and multi‐protein DNA replication complexes in the chromatophore that all rely on nuclear‐encoded proteins (containing crTPs) to complete critical steps in these pathways (Gabr, Stephens, & Bhattacharya, 2022).

### The metabolite transporter conundrum

Despite the clear need for intermediate metabolites in the KR01 CO_2_ delivery system to cross chromatophore membranes, there is until now, no evidence of such transporters in *Paulinella*. Only the BASS2/NHD/PPT system (pyruvate and phosphoenolpyruvate transport) and ACC (ADP/ATP) transporters have been identified in KR01, and most of the genes encoding these proteins show significant upregulation under HL (Table 2). Their response to elevated CO_2_ is much more variable with only some showing downregulation under BC and HLBC relative to HL treatments, as expected if they supported a CCM. However, most of these genes were non‐significantly up or downregulated. Based on the conditions examined here, light regulation of transporters appears to be stronger than CO_2_ regulation. Previous work has documented the substantial loss of chromatophore functions due to genome reduction, whereby all existing transporters were lost (Nowack et al., 2008). Given the absence of DiT1/DiT2 transporters in the nuclear genome, *Paulinella* may be using novel/unknown transporters, or some other mechanism to transport malate, OAA, and aspartate across the chromatophore membrane. The capacity of *Paulinella* to transport metabolites into and out of the chromatophore remains to be better understood with the current data suggesting that proteins related to octotricopeptide repeats modulate membrane permeability (Oberleitner et al., 2020), potentially in a non‐specific manner. Therefore, despite the interesting patterns of differential gene expression described here, the transport of intermediates (or other metabolites) in photosynthetic *Paulinella* species needs to be studied in greater detail before the data can be interpreted with confidence and their potential roles in the putative CCM validated.

**TABLE 2:** Differential accumulation of transcripts encoding metabolite transporters in KR01.

Type	Name	KR01 protein ID	Log_2_fold change			
			BC	HL	HLBC	pH
BASS2	Sodium/pyruvate cotransporter BASS2	g3409.t1	−0.462596886	0.44055204	−0.199370534	0.023397953
		g36985.t1	0.342537197	0.572737556	0.055182783	−1.761687937
		g6017.t1	0.208040665	2.368170548	0.220302585	0.68073465
		g9846.t1	−0.105604452	0.271960266	−0.157725036	−0.931268941
NHD	Sodium/proton antiporter	g34532.t1	0.168671398	1.259598084	0.096654542	−0.028242218
PPT	Phosphoenolpyruvate/phosphate translocator	g83363.t1	0.209005065	0.242820117	−0.013510984	−0.349191937
		g67070.t1	0.546147721	−0.486474954	0.007120058	−0.752314277
ACC	ADP/ATP translocase	g38364.t1	−0.908874292	1.075419873	−0.553077921	−0.986137332
		g19135.t1	−0.964430413	0.639962774	−0.833825505	−1.96556924
		g10874.t1	0.460129733	1.276002128	0.145785552	−0.950788843
		g12080.t1	−0.235413781	0.419403229	−0.51164345	−0.768198564
		g27810.t1	−0.519261773	−0.558744165	−0.813336744	−0.55077995
		g23146.t1	−0.124365416	3.085761845	0.203004243	−0.25154044
		g35605.t1	−0.907720069	0.768510981	−0.005188857	−0.005993943
		g22806.t1	0.605537996	0.589901006	0.489717614	−1.972759173
		g65564.t1	−1.22723981	0.158106945	−0.777759135	−0.923500407
		g27044.t1	0.045401292	0.013352215	−0.451370074	−0.55077995

*Note*: Significantly (*p*‐adjusted <0.05) upregulated (orange) and downregulated (blue) genes are highlighted.

### An alternative model for the putative Paulinella CCM

An open question is: why does the slow‐growing *Paulinella* need to increase CO_2_ delivery to the chromatophore via a putative biochemical CCM? In this regard, Marin et al. (2007) suggested that the intracellular environment in *P. chromatophora* may be CO_2_‐rich due to host respiration, and therefore an efficient CCM may not be needed in this species. However, this was conjecture, and they noted the need for experimental data to address this issue. Perhaps more interesting is the observation that these amoebae cannot tolerate even moderate light levels, which suggests that CO_2_ limitation may not be constraining photosynthesis. Such light sensitivity is, however, expected in a novel photosynthetic lineage that must manage toxic waste, such as reactive oxygen produced by the photosynthetic electron transport system. For photosynthetic *Paulinella*, slow growth does not appear to be explained by a poorly functioning photosynthesis machinery (Gabr, Zournas, et al., 2022). Rather, this phenotype is most likely explained by how the host amoeba partitions photosynthesis‐derived fixed carbon and reductant and manages photosynthesis‐derived waste products (e.g., reactive oxygen species) generated by the efficient cyanobacterial endosymbiont electron transport system (Stephens et al., 2021). We refer to this scenario as the “chassis and engine” model (Stephens et al., 2021) that proposes that the *Paulinella* host (not the endosymbiont) is under strong selective pressure to evolve novel traits, relative to the heterotrophic ancestor, that may facilitate the continued integration of the chromatophore into host metabolism. It is therefore possible that the different photosynthetic *Paulinella* hosts evolved (or more likely, are evolving) novel CO_2_ delivery systems to reduce the accumulation of toxins that arise from both the oxygenation activity of RuBisCO, which is likely compounded by a seemingly nascent and inefficient C_2_ photorespiratory cycle, and the suboptimal export of photosynthetic products from their chromatophores. In both cases, natural selection appears to favour the low light‐adapted, slow‐growth phenotype that ensures the survival of *Paulinella* in aquatic habitats dominated by phototrophs that contain the highly efficient Archaeplastida‐derived plastid (e.g., diatoms, dinoflagellates). An “inefficient” CO_2_ delivery system may have evolved to allow *Paulinella* to balance the low levels of O_2_ production in low‐light habitats with the acquisition of CO_2_ needed for carbon fixation, thereby minimizing energetic losses due to photorespiration. This novel trait, which has persisted for millions of years, may also allow *Paulinella* to survive during times of emersion or partial desiccation, given that a putative novel strain/species was recently described that exists as an epiphyte on the roots of aquatic grasses (Van Etten et al., 2023).

The multiple independent origins of different types of CCMs in microalgae suggest that selection to increase CO_2_ concentration in the vicinity of RuBisCO is a powerful driving force in the evolution of photosymbionts (Kupriyanova et al., 2023; Wei et al., 2019). The complex and incomplete (e.g., metabolite transporters) results we report here for KR01 likely indicate that the putative biochemical CCM in this lineage is still evolving and does not have the hallmarks of canonical CO_2_ delivery pathways in algae and plants, whose ancestor originated >1.6 Bya. Given this uncertainty, the path forward with *Paulinella* to test our hypotheses is to use approaches such as proteomics to validate the RNA‐seq results, immunological approaches to determine the subcellular locations of the putative CCM components, metabolomic profiling, and ^13^CO_2_ tracing to determine if changes in the expression of putative CCM‐related genes correspond with a predictable shift in metabolite pools. Physiological studies with low CO_2_‐acclimated cultures are needed to examine whether carbon fixation is indeed supported by the production and intracellular transport of metabolites arising from the novel CCM and to quantify predicted C_2_ cycle bottlenecks that may limit the efficiency of photorespiration. Experimental validation of the subcellular localization of these proteins is also needed to determine the extent of compartmentalization of these functions in *Paulinella*.

## AUTHOR CONTRIBUTIONS

**Arwa Gabr:** Writing – review and editing (equal). **Timothy G. Stephens:** Writing – review and editing (equal). **John R. Reinfelder:** Writing – review and editing (equal). **Pinky Liau:** Writing – review and editing (equal). **Victoria Calatrava:** Writing – review and editing (equal). **Arthur R. Grossman:** Writing – review and editing (equal). **Debashish Bhattacharya:** Conceptualization (equal); supervision (equal); writing – review and editing (equal).

## CONFLICT OF INTEREST STATEMENT

The authors declare no conflicts of interest.

## Supporting information


**Supplemental Dataset 1.** Phylogenetic trees of the PEPC, AST, MDH, ALT, PCK, MAEB, and PPDK proteins identified in KR01 along with protein sequences retrieved using BLASTp from a taxonomically broad local database. Node support values are based on 2000 ultrafast bootstrap approximations; nodes with support ≥95% are annotated with black circles. Sequence labels are coloured based on the taxonomic domain of the organism; the internal branches of monophyletic clades are coloured the same as their members, and polyphyletic clades are coloured black. The legend in the top left describes the colours and shapes used in the figure.

**Supplemental Dataset 2.** Individual phylogenetic trees of the KR01 proteins annotated as MDH (g8927.t1, g55981.t1, g62328.t1, and g43748.t1) and PCK (g43353.t1 and g42094.t1) enzymes along with protein sequences retrieved using BLASTp from a taxonomically broad local database. Node support values are based on 2000 ultrafast bootstrap approximations; nodes with support ≥95% are annotated with black circles. Sequence labels are coloured based on the taxonomic domain of the organism; the internal branches of monophyletic clades are coloured the same as their members, and polyphyletic clades are coloured black. The legend in the top left describes the colours and shapes used in the figure.

**Supplemental Figure 1.** RNA‐seq sample‐level QC. (A) principal component analysis (PCA) of samples coloured by treatment using DESeq2 normalized counts. Open black circles encapsulate sample outliers that were removed from downstream analysis. (B) Heatmap of the pairwise correlation values between each combination of samples using DESeq2 normalized counts. Graphs were constructed RStudio 2021.9.2.382 using the DESeq2 package. Treatments: HL, high light; BC, 5 mM bicarbonate; HLBC, high light and 5 mM bicarbonate; pH, pH = 8.0.
**Supplemental Figure 2.** Venn diagram showing the number of DEGs that are unique and shared among the four treatments. The number in parenthesis next to each treatment label represents the total number of significant (adjusted *p*‐value <0.05) DEGs. The figure was generated using the Venn Diagram tool from OmicBox 2.0.36.
**Supplemental Figure 3.** Top 30 GO terms ordered by Fisher exact *p*‐value. TopGO analysis of significantly (adjusted *p*‐value <0.05) DEGs from different treatments: (A) HL, high light; (B) BC, 5 mM bicarbonate; (C) HLBC, high light and 5 mM bicarbonate; (D) pH, pH = 8.0.

**Supplemental Table 1.** RNA‐seq data from all treatment replicates.
**Supplemental Table 2.** Subcellular localization analysis of putative biochemical CCM‐related proteins in KR01 using different prediction programs.
**Supplemental Table 3.** RNA‐seq raw read count for transcript expression in control and different treatment groups.
**Supplemental Table 4.** DESeq2 HL versus Control output with KEGG, GO, and BLAST annotation. Highlighted cells represent significantly differentially expressed genes (*p*‐adjusted <0.05).
**Supplemental Table 5.** DESeq2 BC versus Control output with KEGG, GO, and BLAST annotation. Highlighted cells represent significantly differentially expressed genes (*p*‐adjusted <0.05).
**Supplemental Table 6.** DESeq2 HLBC versus Control output with KEGG, GO, and BLAST annotation. Highlighted cells represent significantly differentially expressed genes (*p*‐adjusted <0.05).
**Supplemental Table 7.** DESeq2 pH versus Control output with KEGG, GO, and BLAST annotation. Highlighted cells represent significantly differentially expressed genes (*p*‐adjusted <0.05).
**Supplemental Table 8.** GO terms enriched in transcripts from HL‐treated cells were compared against all transcripts that aligned to the KR01 genome.
**Supplemental Table 9.** GO terms enriched in transcripts from BC‐treated cells were compared against all transcripts that aligned to the KR01 genome.
**Supplemental Table 10.** GO terms enriched in transcripts from HLBC‐treated cells were compared against all transcripts that aligned to the KR01 genome.
**Supplemental Table 11.** GO terms enriched in transcripts from pH‐treated cells were compared against all transcripts that aligned to the KR01 genome.

## Data Availability

RNA‐seq data from KR01 are available from the NCBI's SRA repository (BioProject ID PRJNA847773). Alignments and trees produced by phylogenetic analysis are available in Zenodo: https://zenodo.org/doi/10.5281/zenodo.6639903.
