# Supplementary material for: Evidence of a putative CO2 delivery system to the chromatophore in the photosynthetic amoeba Paulinella
Source: Environ Microbiol Rep. 2024 Jun 23;16(3):e13304. doi: 10.1111/1758-2229.13304 (PMC11194058; doi:10.1111/1758-2229.13304)
Supplement: Supplementary file 3 — Supplemental Figure 1. RNA‐seq sample‐level QC. (A) principal component analysis (PCA) of samples coloured by treatment using DESeq2 normalized counts. Open black circles encapsulate sample outliers that were removed from downstream analysis. (B) Heatmap of the pairwise correlation values between each combination of samples using DESeq2 normalized counts. Graphs were constructed RStudio 2021.9.2.382 using the DESeq2 package. Treatments: HL, high light; BC, 5 mM bicarbonate; HLBC, high light and 5 mM bicarbonate; pH, pH = 8.0. Supplemental Figure 2. Venn diagram showing the number of DEGs that are unique and shared among the four treatments. The number in parenthesis next to each treatment label represents the total number of significant (adjusted p‐value <0.05) DEGs. The figure was generated using the Venn Diagram tool from OmicBox 2.0.36. Supplemental Figure 3. Top 30 GO terms ordered by Fisher exact p‐value. TopGO analysis of significantly (adjusted p‐value <0.05) DEGs from different treatments: (A) HL, high light; (B) BC, 5 mM bicarbonate; (C) HLBC, high light and 5 mM bicarbonate; (D) pH, pH = 8.0. [file EMI4-16-e13304-s001.pdf]

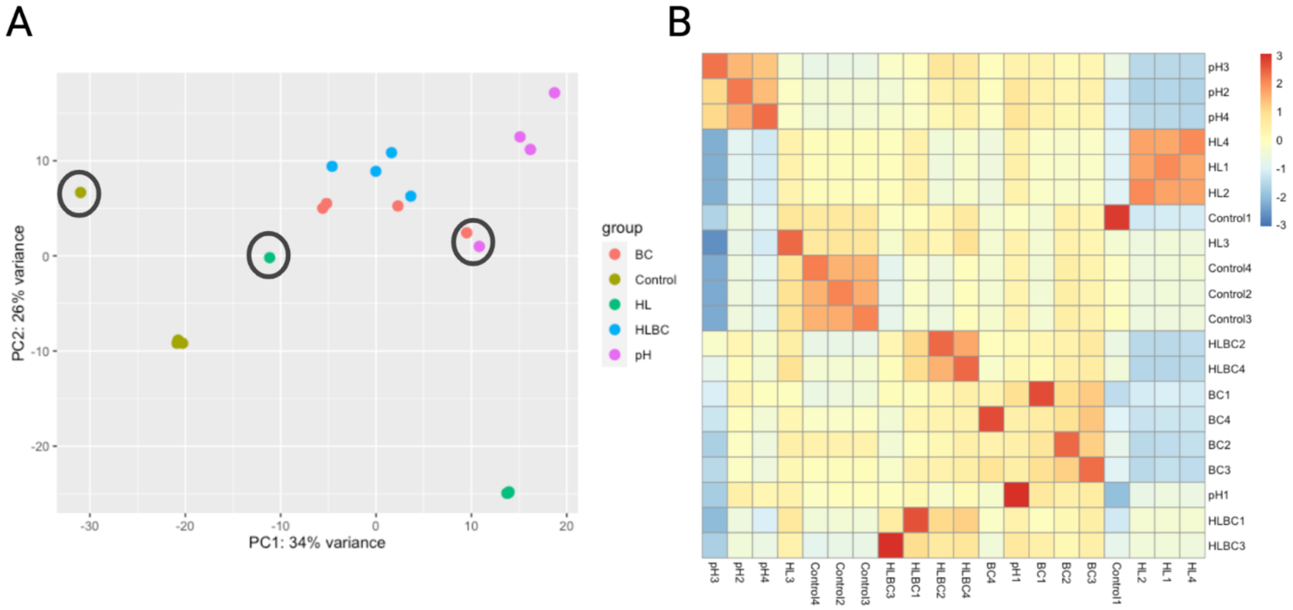

Supplemental Figure 1: RNA-seq sample-level QC. (A) principal component analysis (PCA) of samples colored by treatment using DESeq2 normalized counts. Open black circles encapsulate sample outliers that were removed from downstream analysis. (B) Heatmap of the pairwise correlation values between each combination of samples using DESeq2 normalized counts. Graphs were constructed RStudio 2021.9.2.382 using the DESeq2 package. Treatments: HL, high light; BC, 5mM bicarbonate; HLBC, high light and 5mM bicarbonate; pH, pH=8.0.

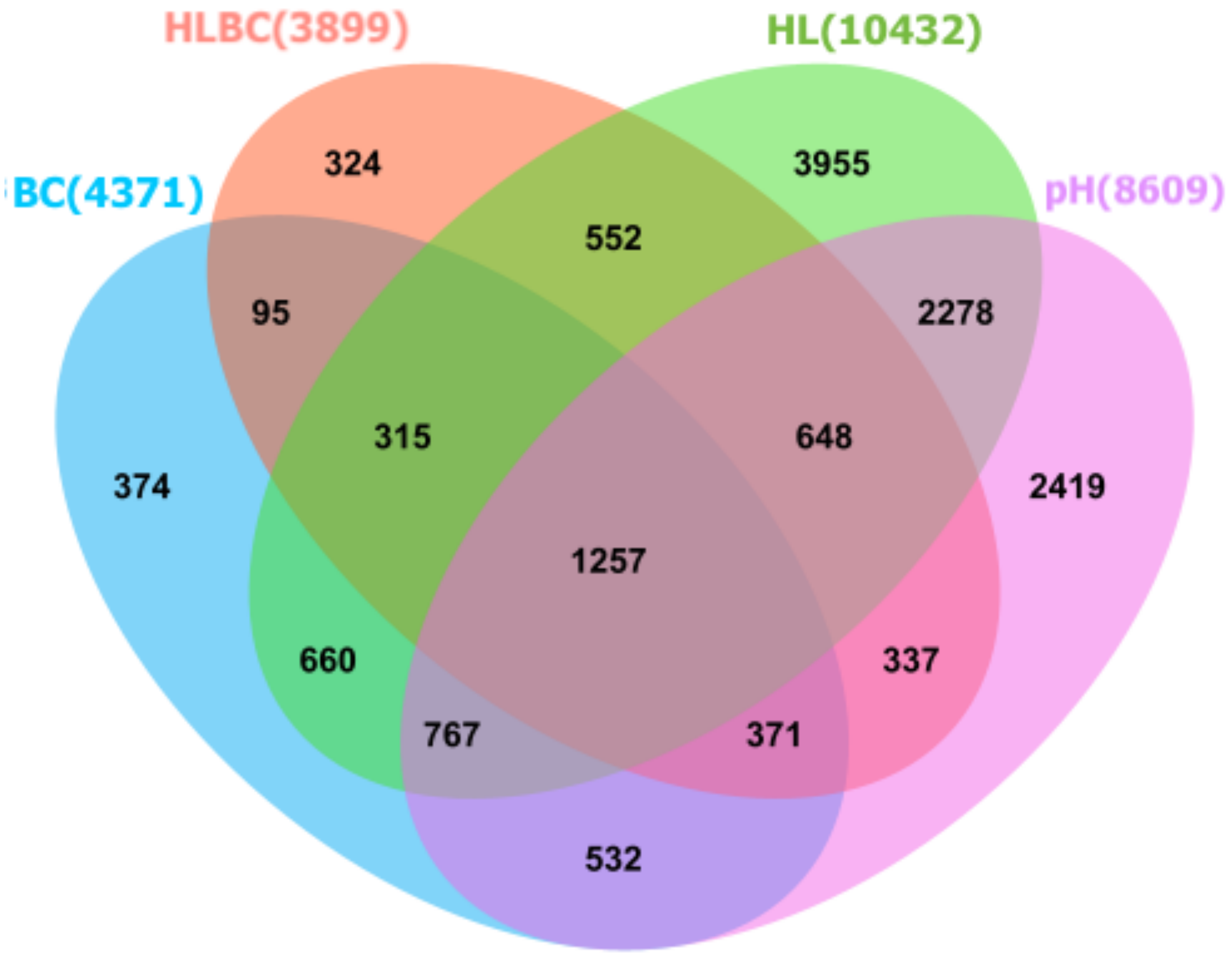

Supplemental Figure 2. Venn diagram showing the number of DEGs that are unique and shared among the four treatments. The number in parenthesis next to each treatment label represents the total number of significantly (adjusted *p*-value < 0.05) DEGs. Figure was generated using the Venn Diagram tool from OmicBox 2.0.36.

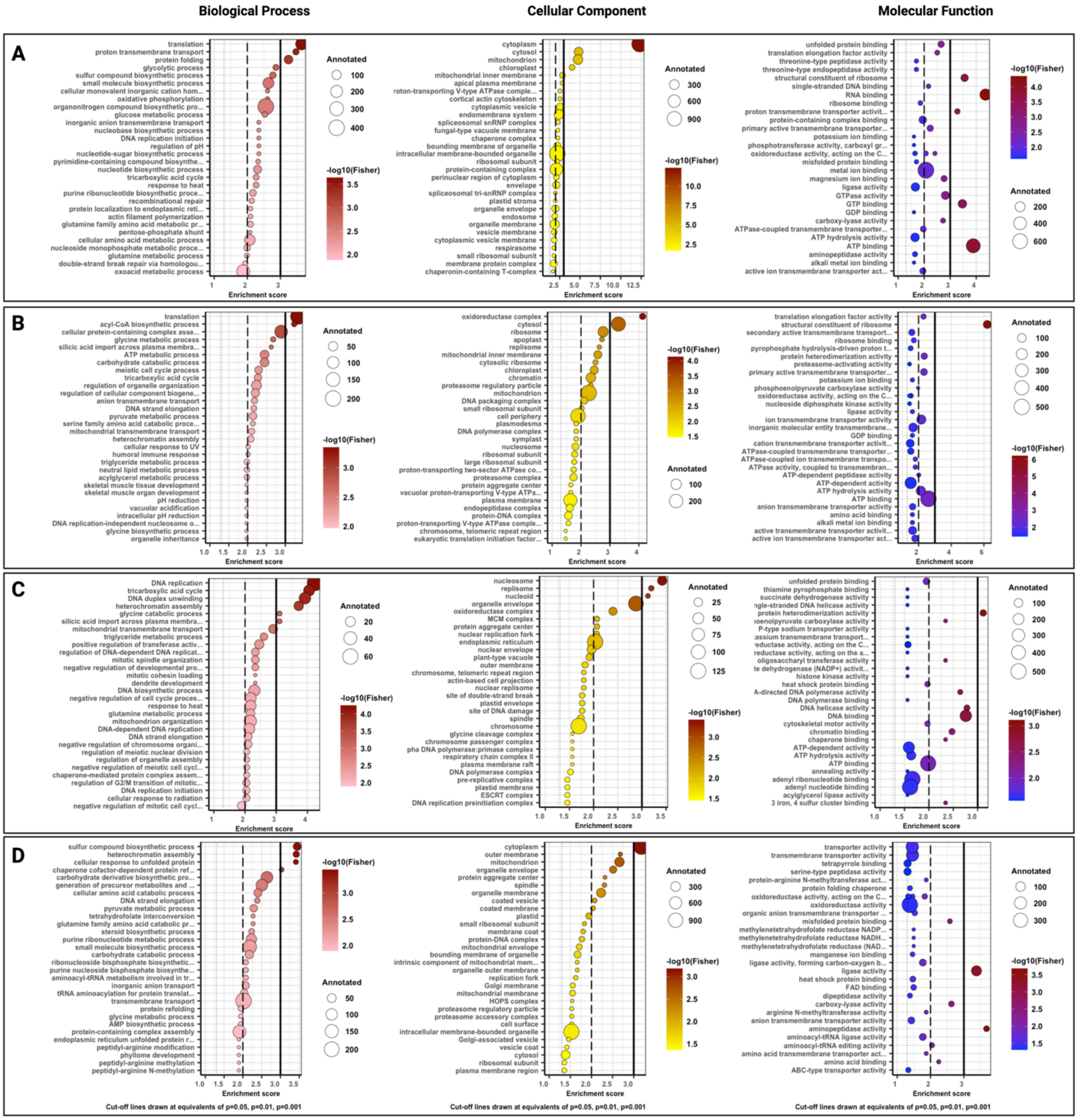

S**upplemental Figure 3.** Top 30 GO terms ordered by Fisher Exact *p*-value. TopGO analysis of significantly (adjusted *p*-value < 0.05) DEGs from different treatments: (A) HL, high light; (B) BC, 5mM bicarbonate; (C) HLBC, high light and 5mM bicarbonate; (D) pH, pH=8.0.

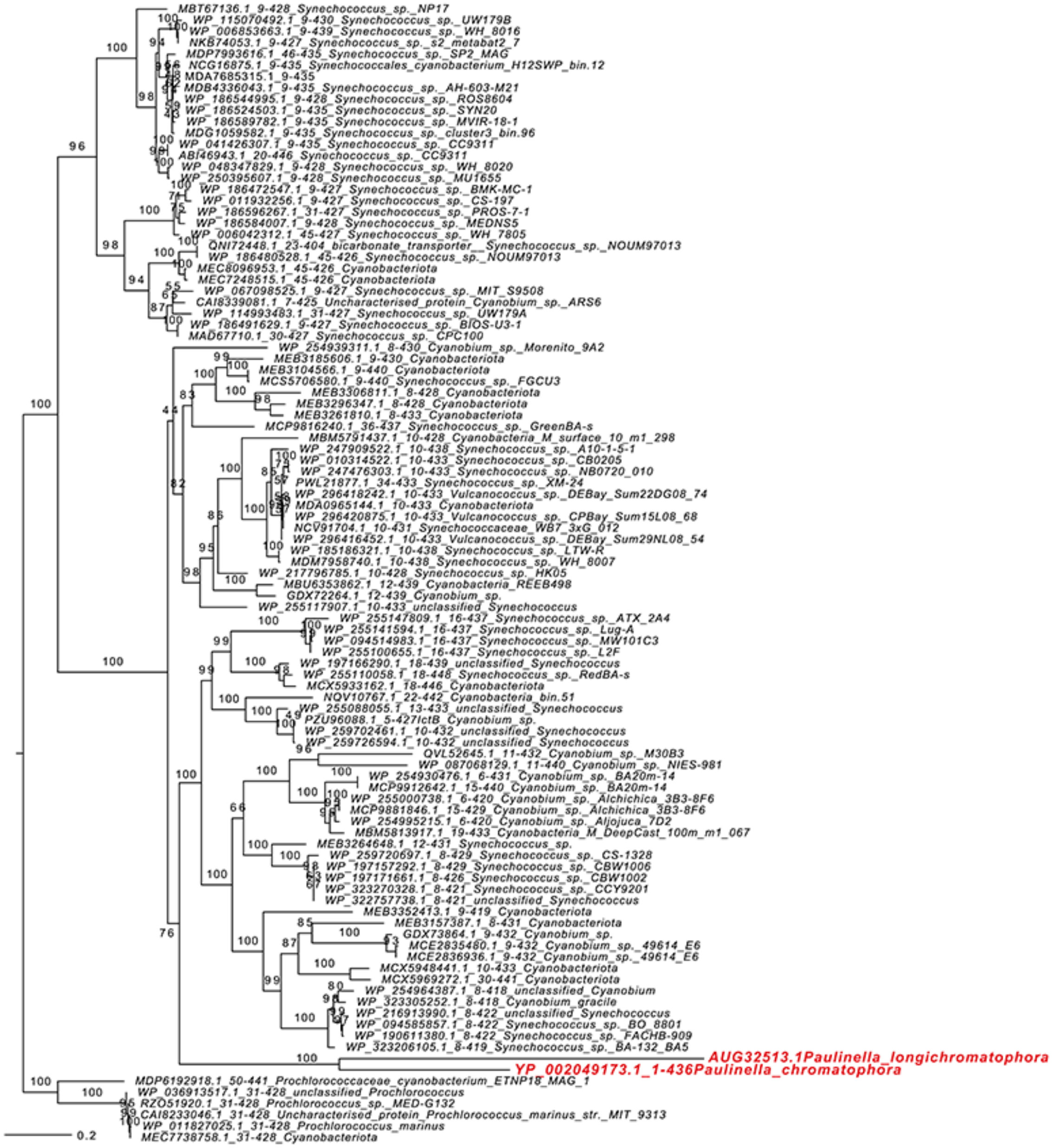

Supplemental Figure 4. Protein maximum likelihood phylogeny of inorganic carbon transporters (*ictB*) from cyanobacteria and *Paulinella* species. The sequences were aligned using MAFFT (default option: --auto; v7.487; Yamada et al. 2016) and the tree built using IQ-tree v1.6.12 (Nguyen et al. 2015) with ultrafast bootstrapping of 1,000 replications (-bb 1000) and model testing (-m TEST).

Yamada, K. D., Tomii, K., & Katoh, K. (2016). Application of the MAFFT sequence alignment program to large data-reexamination of the usefulness of chained guide trees. Bioinformatics. 32(21), 3246–3251. doi:10.1093/bioinformatics/btw412

Nguyen, L. T., Schmidt, H. A., von Haeseler, A., & Minh, B. Q. (2015). IQ-TREE: a fast and effective stochastic algorithm for estimating maximum-likelihood phylogenies. Mol Biol Evol. 32(1), 268–274. doi:10.1093/molbev/msu300
