# Supplementary material for: Evidence of a putative CO2 delivery system to the chromatophore in the photosynthetic amoeba Paulinella
Source: Environ Microbiol Rep. 2024 Jun 23;16(3):e13304. doi: 10.1111/1758-2229.13304 (PMC11194058; doi:10.1111/1758-2229.13304)

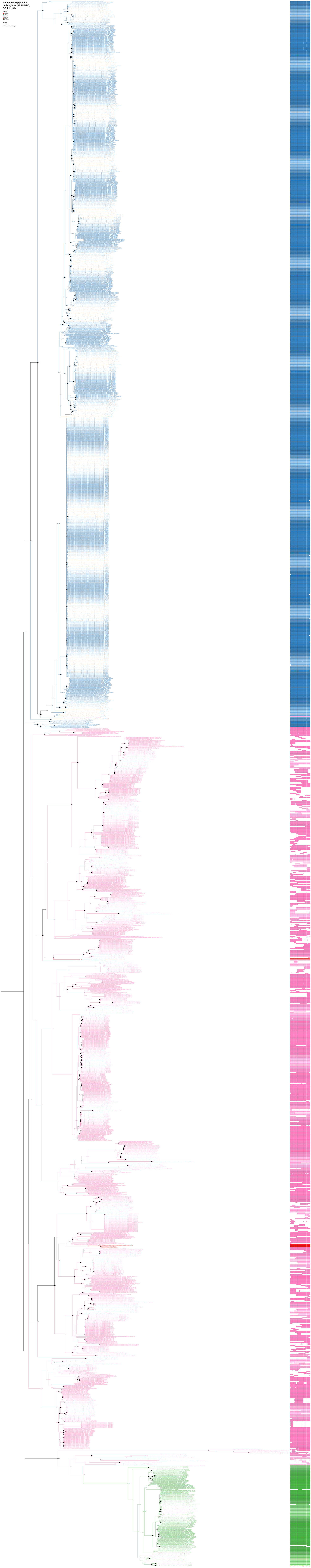

Phosphoenolpyruvate carboxylase (PEPC/PPC; EC 4.1.1.31)
Groups
Paulinella
Archaea
Bacteria
Eukaryota
Viruses
Other/Misc
Nodes
BS ≥ 95%
BS: ultrafast bootstrap support

Aspartate aminotransferase (AST; EC 2.6.1.1)

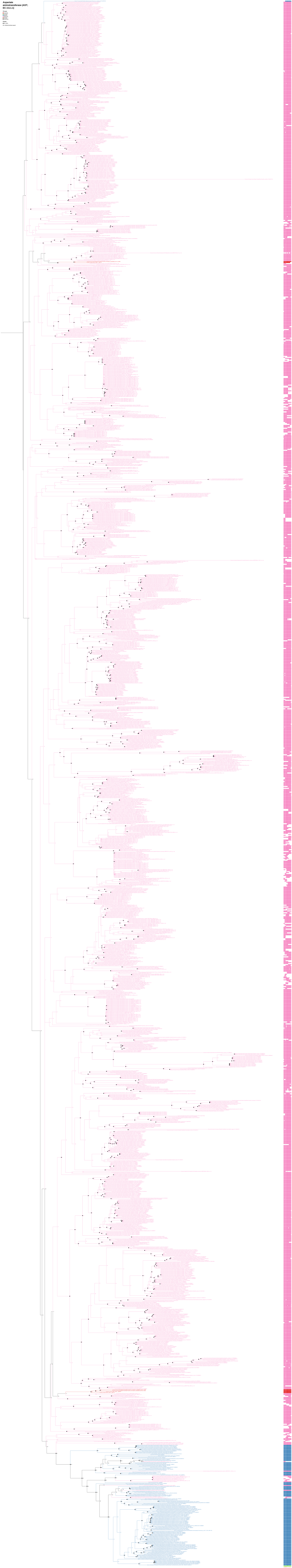

Malate dehydrogenase (MDH; EC 1.1.1.37)

Groups
- *Paulinella*
- Archaea
- Bacteria
- Eukaryota
- Viruses
- Other/Misc

Nodes
- ● *BS* ≥ 95%

BS: ultrafast bootstrap support

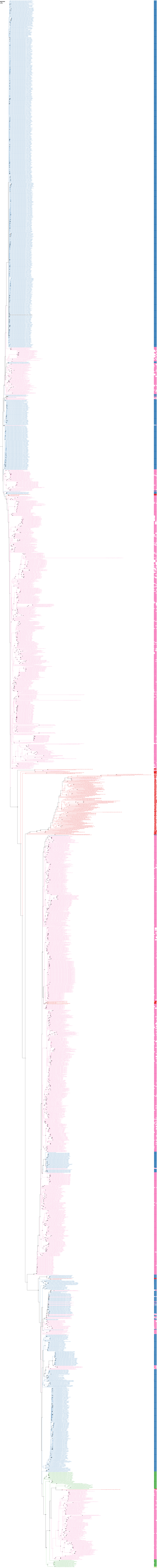

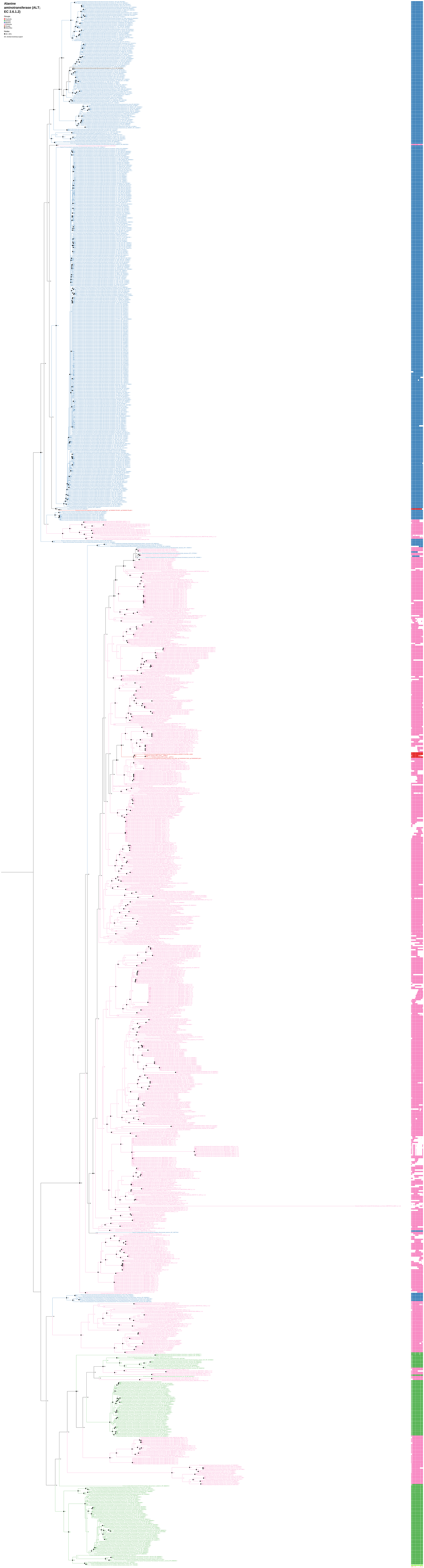
Alanine aminotransferase (ALT; EC 2.6.1.2)
Groups
Paulinella
Archaea
Bacteria
Eukaryota
Viruses
Other/Misc
Nodes
BS ≥ 95%
BS: ultrafast bootstrap support

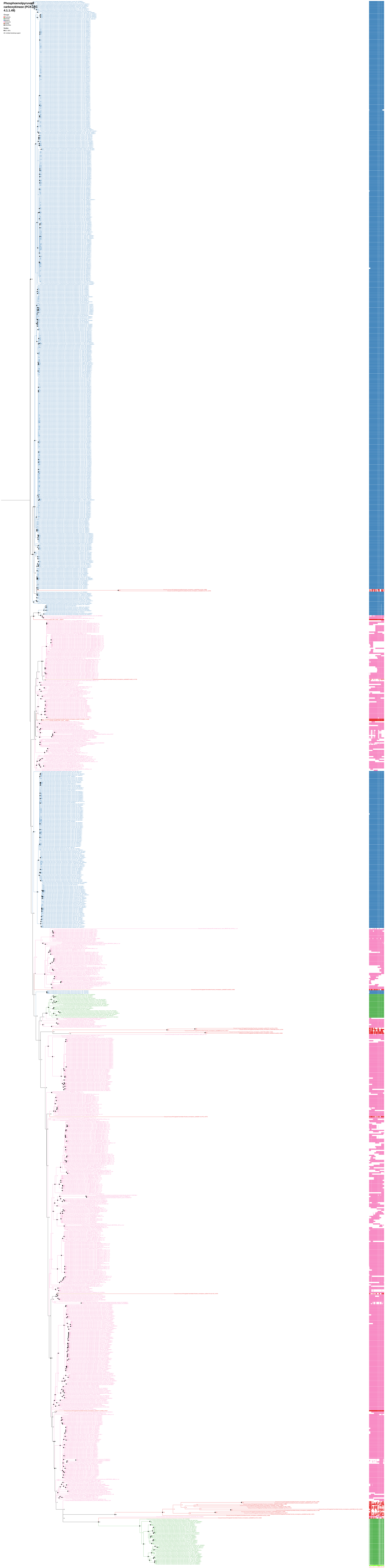
Phosphoenolpyruvate carboxykinase (PCK; EC 4.1.1.49)
Groups
Paulinella
Archaea
Bacteria
Eukaryota
Viruses
Other/Misc
Nodes
BS ≥ 95%
BS: ultrafast bootstrap support

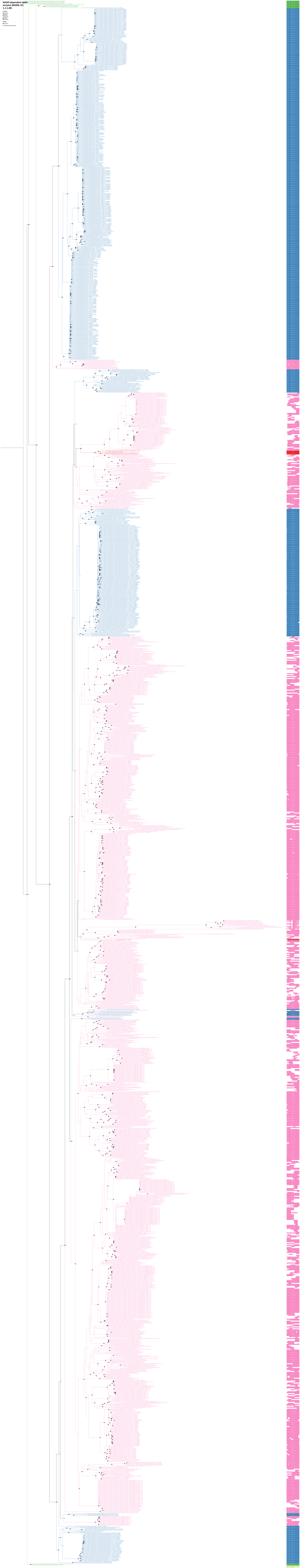

NADP-dependent malic enzyme (MAEB; EC 1.1.1.40)
Groups
Paulinella
Archaea
Bacteria
Eukaryota
Viruses
Other/Misc
Nodes
BS ≥ 95%
BS: ultrafast bootstrap support

Pyruvate, phosphate dikinase (PPDK; EC 2.7.9.1)

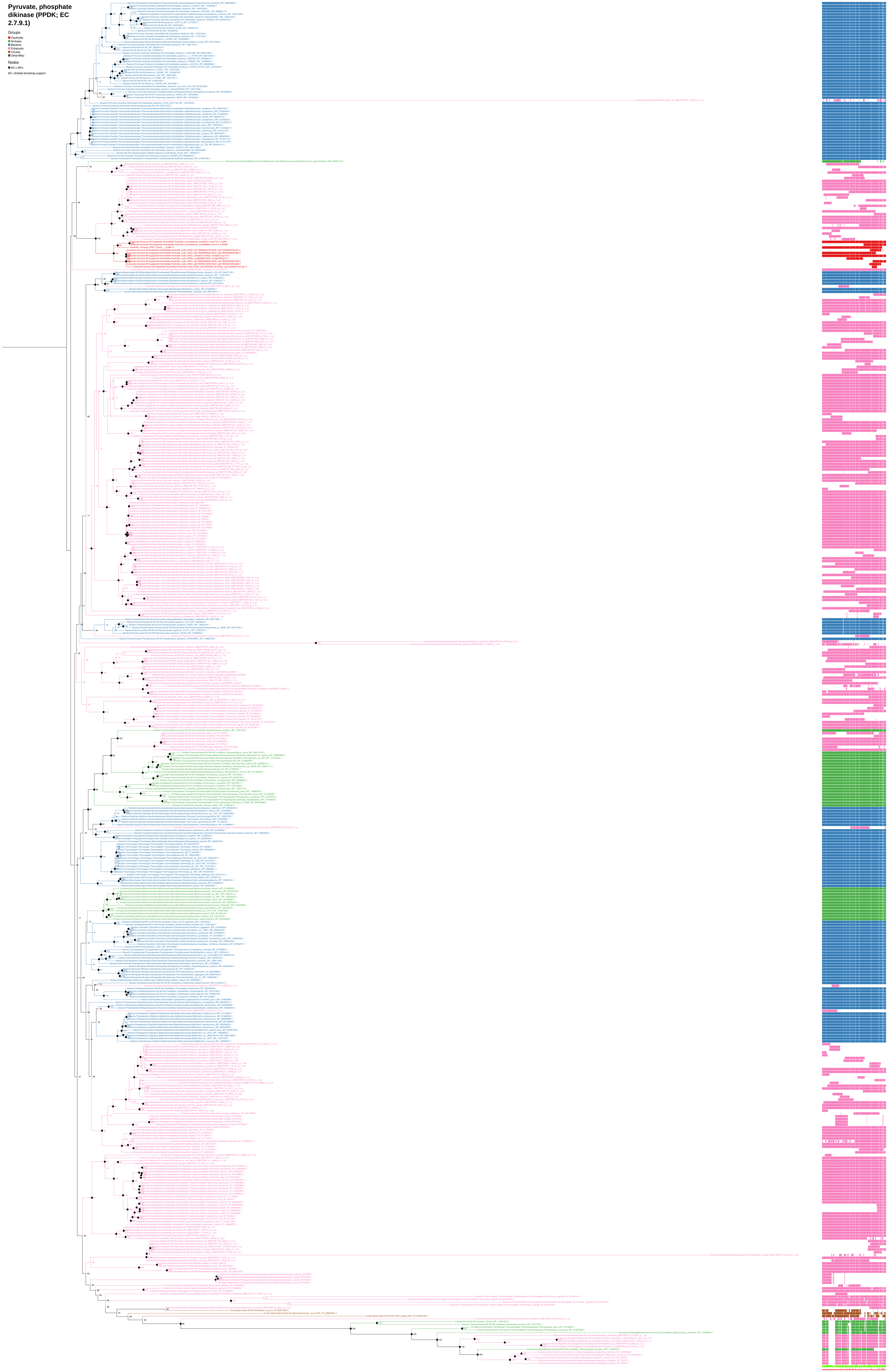

Supplement: Supplementary file 1 — Supplemental Dataset 1. Phylogenetic trees of the PEPC, AST, MDH, ALT, PCK, MAEB, and PPDK proteins identified in KR01 along with protein sequences retrieved using BLASTp from a taxonomically broad local database. Node support values are based on 2000 ultrafast bootstrap approximations; nodes with support ≥95% are annotated with black circles. Sequence labels are coloured based on the taxonomic domain of the organism; the internal branches of monophyletic clades are coloured the same as their members, and polyphyletic clades are coloured black. The legend in the top left describes the colours and shapes used in the figure. [file EMI4-16-e13304-s004.pdf]
