# Supplementary material for: Evidence of a putative CO2 delivery system to the chromatophore in the photosynthetic amoeba Paulinella
Source: Environ Microbiol Rep. 2024 Jun 23;16(3):e13304. doi: 10.1111/1758-2229.13304 (PMC11194058; doi:10.1111/1758-2229.13304)

g8927.t1 (MDH; Malate dehydrogenase)

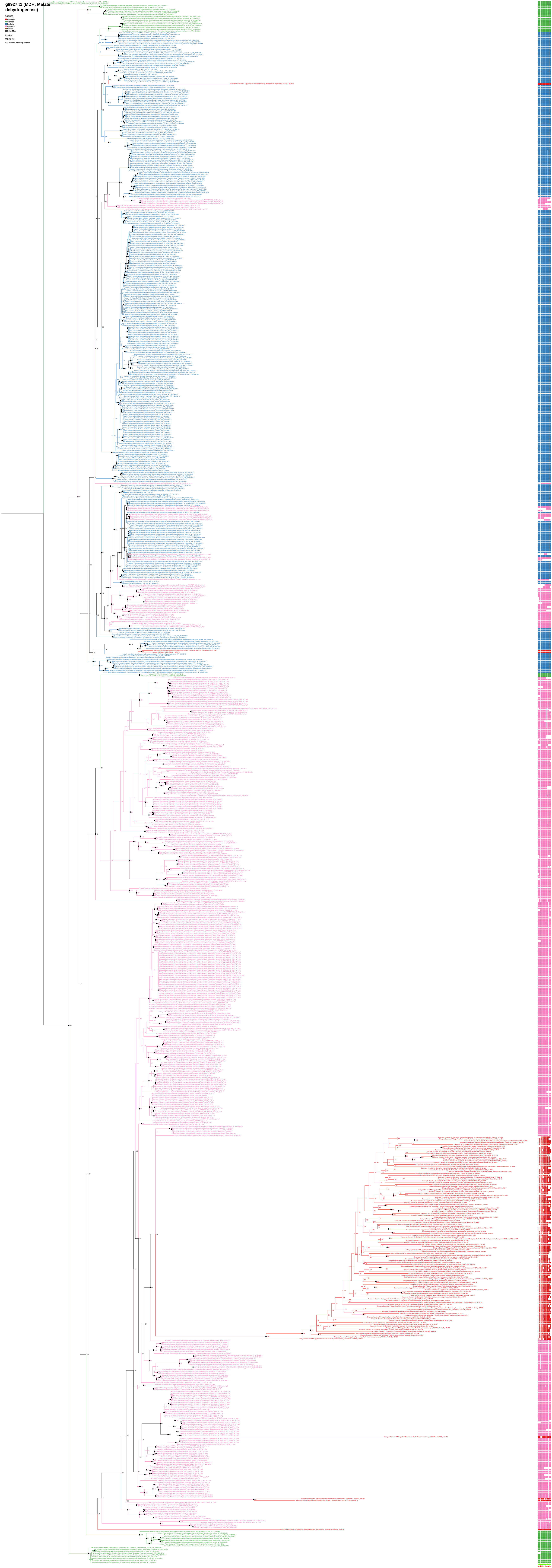

g55981.t1 (MDH; Malate dehydrogenase)

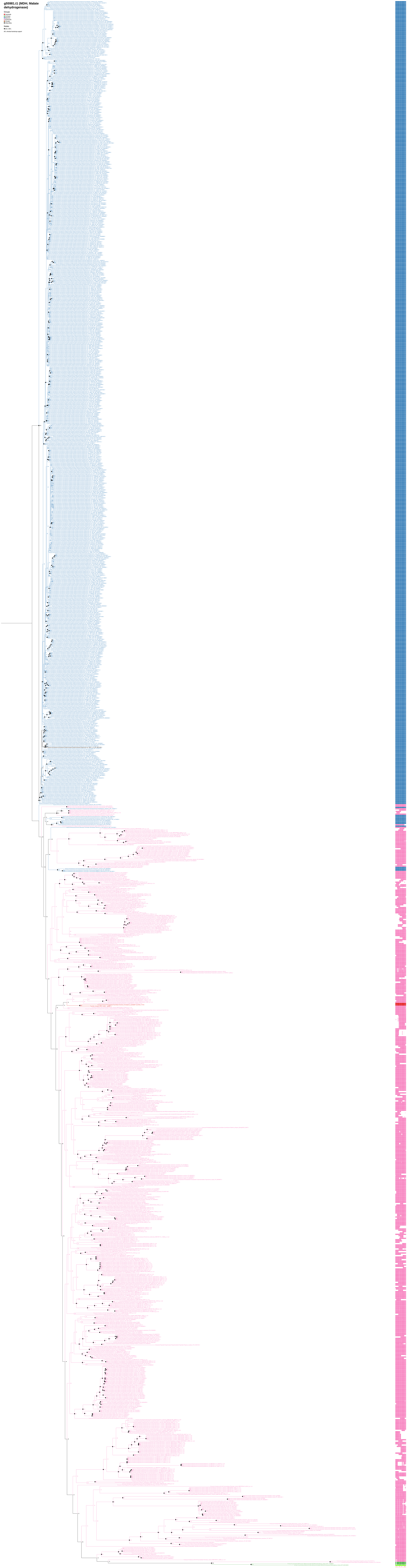

g62328.t1 (MDH; Malate dehydrogenase)

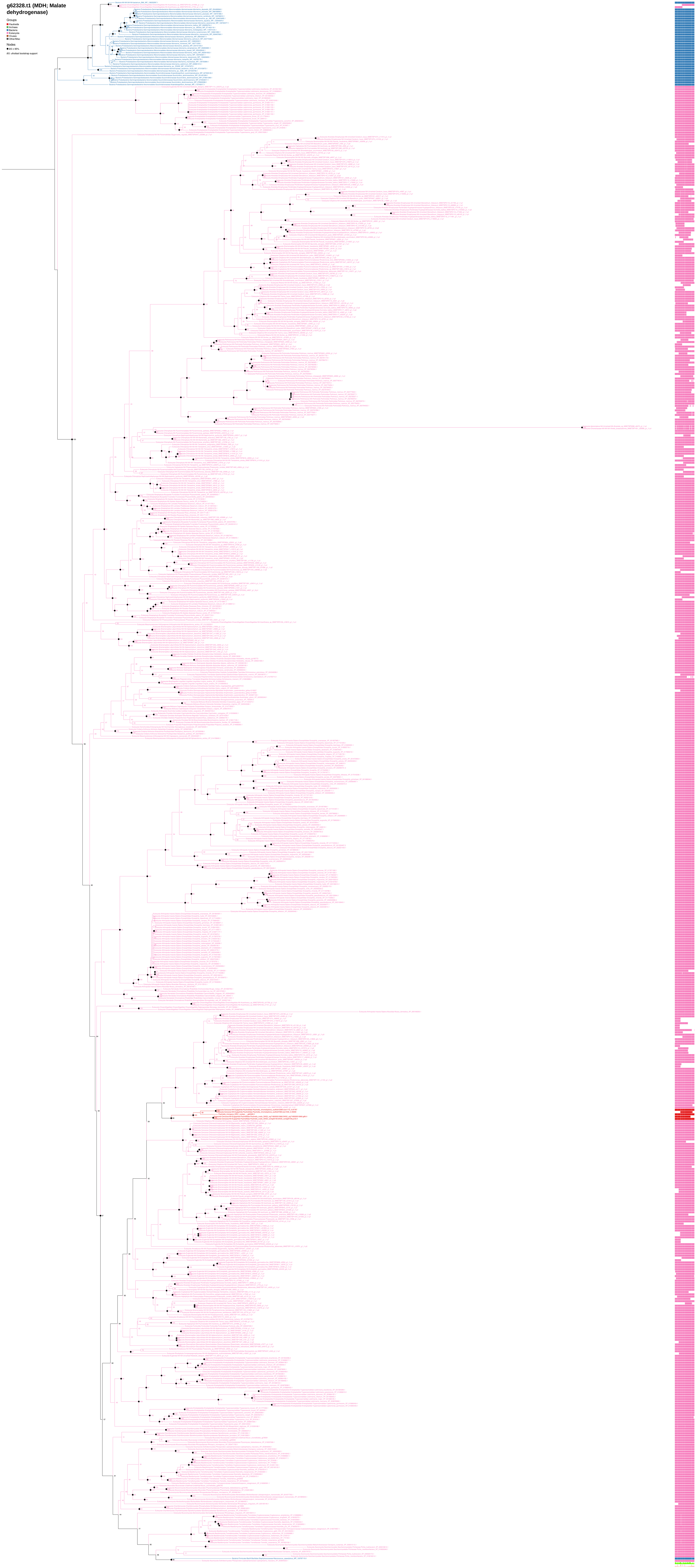

g43748.t1 (MDH; Malate dehydrogenase)

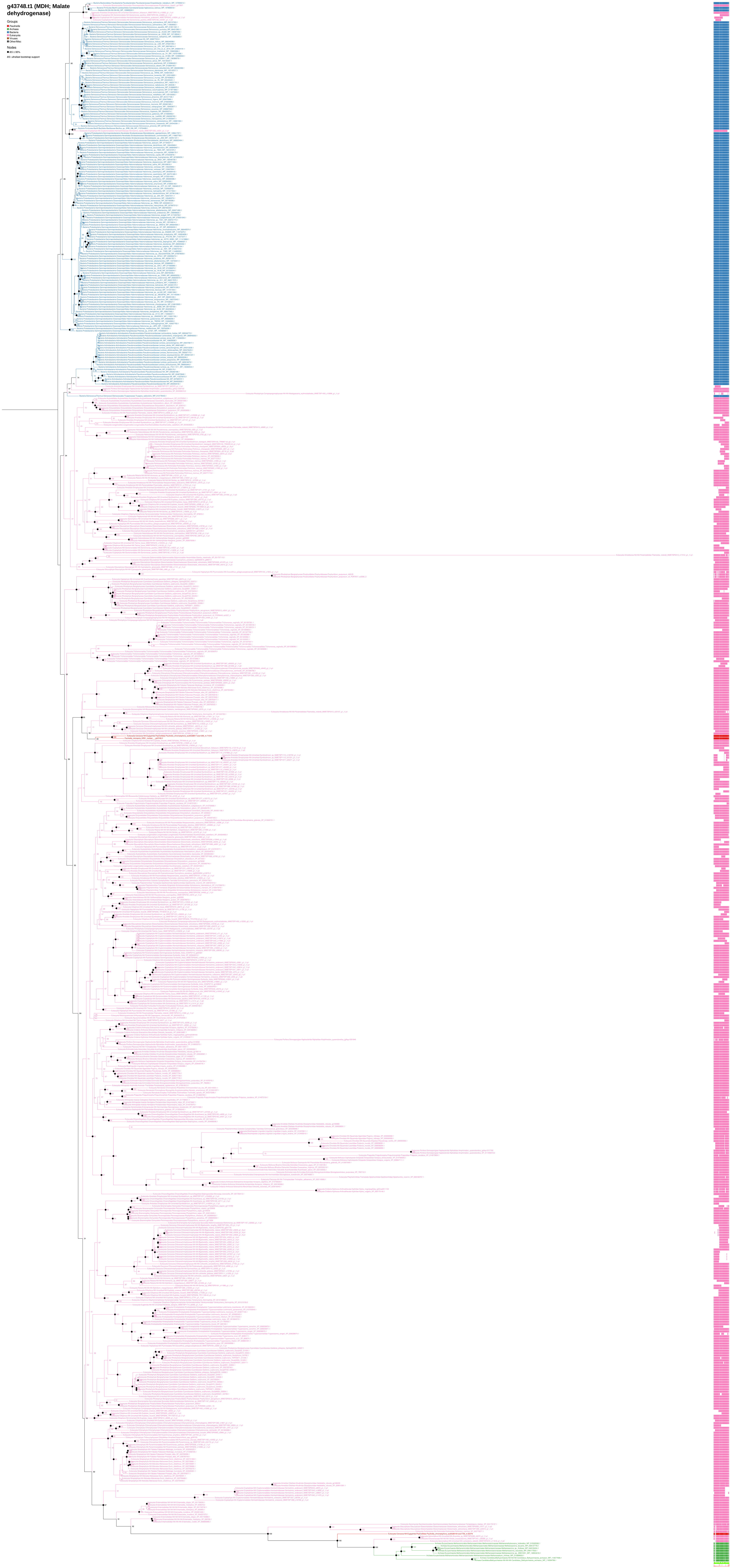

g43353.t1 (PCK; Phosphoenolpyruvate carboxykinase)

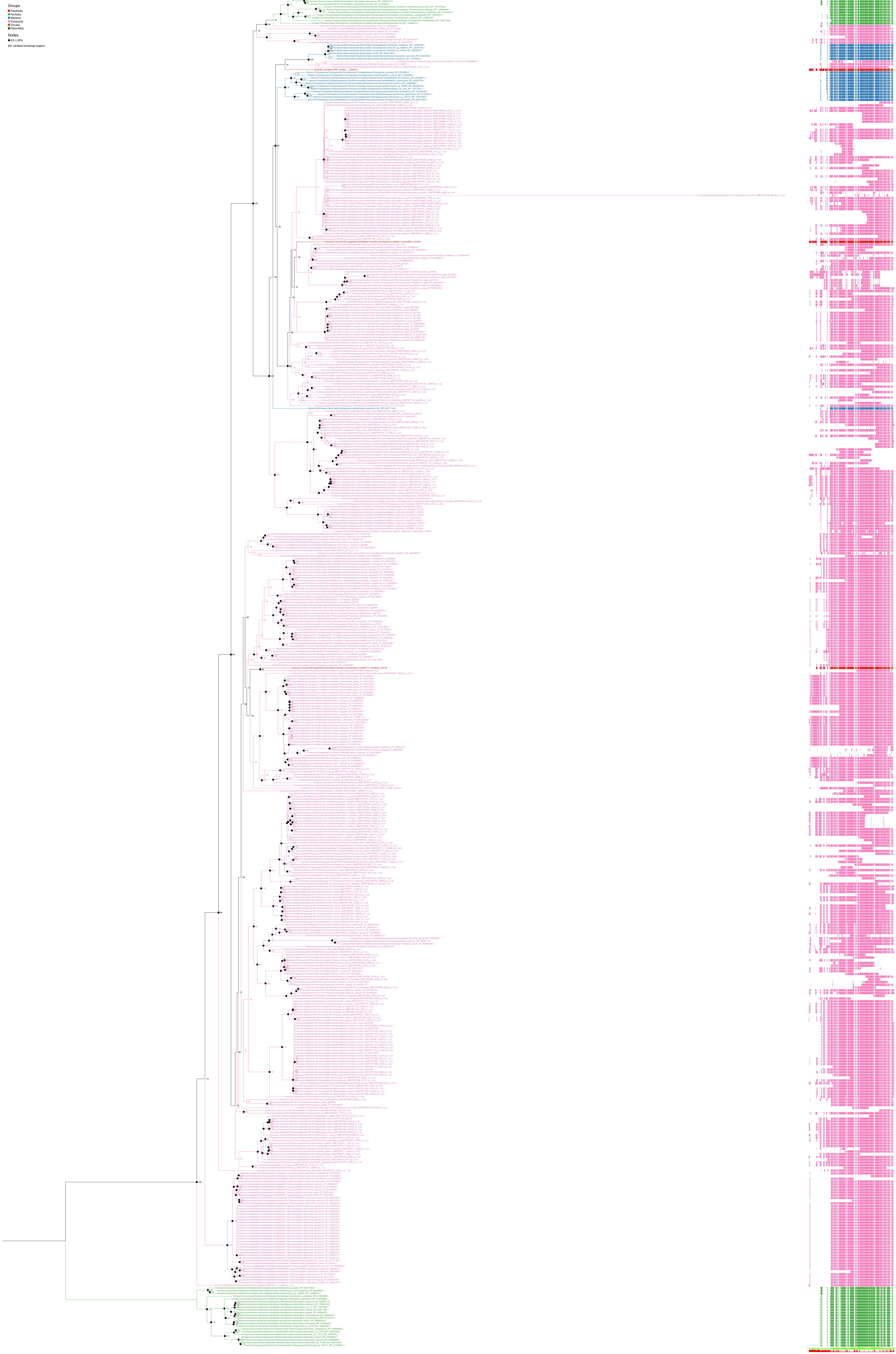

g42094.t1 (PCK; Phosphoenolpyruvate carboxykinase)

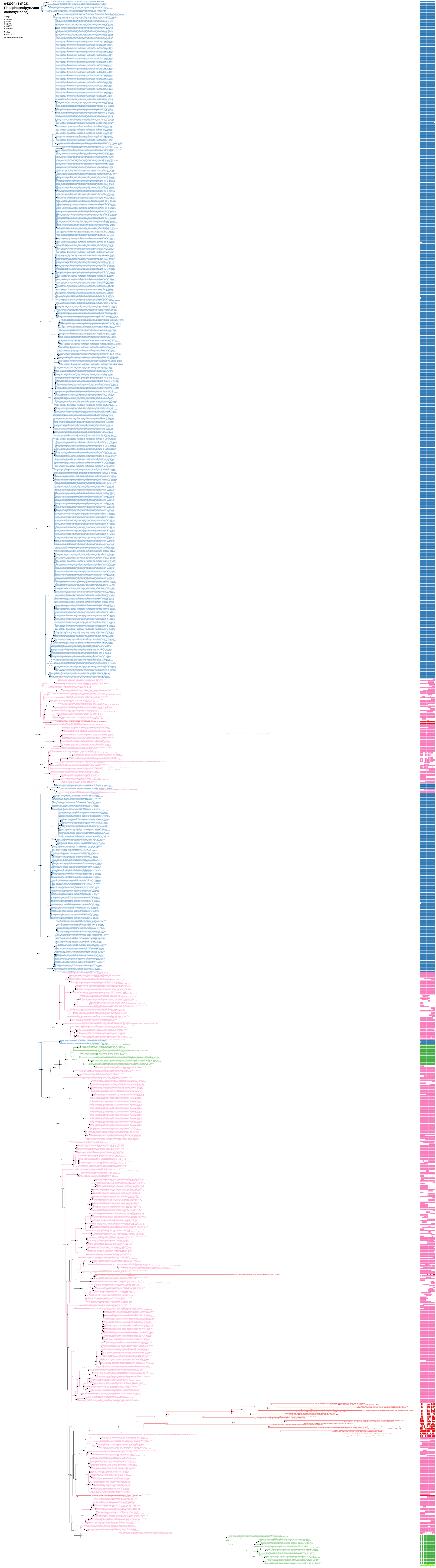

Supplement: Supplementary file 2 — Supplemental Dataset 2. Individual phylogenetic trees of the KR01 proteins annotated as MDH (g8927.t1, g55981.t1, g62328.t1, and g43748.t1) and PCK (g43353.t1 and g42094.t1) enzymes along with protein sequences retrieved using BLASTp from a taxonomically broad local database. Node support values are based on 2000 ultrafast bootstrap approximations; nodes with support ≥95% are annotated with black circles. Sequence labels are coloured based on the taxonomic domain of the organism; the internal branches of monophyletic clades are coloured the same as their members, and polyphyletic clades are coloured black. The legend in the top left describes the colours and shapes used in the figure. [file EMI4-16-e13304-s002.pdf]
